# Effect of sequentially fed high protein, hydrolyzed protein, and high fiber diets on the fecal microbiota of healthy dogs: a cross-over study

**DOI:** 10.1186/s42523-021-00101-8

**Published:** 2021-06-11

**Authors:** Lina María Martínez-López, Amy Pepper, Rachel Pilla, Andrew P. Woodward, Jan S. Suchodolski, Caroline Mansfield

**Affiliations:** 1 Department of Veterinary Clinical Sciences, Melbourne Veterinary School, The University of Melbourne, Werribee, VIC 3030 Australia; 2 Veterinary Specialists of Sydney, Miranda, NSW 2228 Australia; 3 Gastrointestinal Laboratory, Department of Small Animal Clinical Sciences, College of Veterinary Medicine and Biomedical Sciences, Texas A&M University, College Station, TX USA; 4 Faculty of Veterinary and Agricultural Sciences, The University of Melbourne, Werribee, VIC 3030 Australia

**Keywords:** Dog microbiota, Diet, High-insoluble fiber, High-protein, Hydrolyzed, Raw meat diets

## Abstract

**Background:**

Dietary content and environmental factors can shape the gut microbiota, and consequently, the way the gut microbiota metabolizes fats, carbohydrates, and proteins, affecting overall health of the host. We evaluated the impact of 3 diets (all meat [raw], high-insoluble fiber dry extruded diet and hydrolyzed protein dry extruded diet) on the gut microbiota of healthy dogs in a cross-over sequential study.

**Results:**

We showed that diet can have an effect on the gut microbiome in dogs, which was influenced by the order of feeding. High-protein (all meat) diets were characterized by an increase in bacteria belonging to the Fusobacteria and Bacteroidetes phyla, whereas a high-insoluble fiber commercial diet correlated with increases in Firmicutes and Actinobacteria phyla. However, the individual dog’s baseline microbiota had the most impact on the magnitude and nature of the changes in response to dietary intervention.

**Conclusion:**

Our results suggest that the dog fecal microbiota is driven by protein and fiber composition to different degrees in individual animals, and targeted modification of these patterns could be useful in the modulation of the gut microbiota in different diseases.

**Supplementary Information:**

The online version contains supplementary material available at 10.1186/s42523-021-00101-8.

## Background

The gut microbiota is essential for maintaining health, as it exerts several beneficial effects on the host, regulates numerous biological pathways; and interacts directly and indirectly with various organs and systems in the body, including the brain, liver, bone, and cardiovascular system [1]. The gut microbiota is a highly complex community that evolves rapidly and adapts to its host over a lifetime and exhibits a remarkable plasticity to environmental changes, particularly diet [2, 3].

Even short-term dietary changes have been shown to alter human gut microbiota composition and changes can be observed within 1–3 days when there are extreme dietary modifications such as switching between an all-meat to an all-plant diet [4]. Similar studies have been performed regarding the effect of fiber on the gut microbiota of dogs [5–15]. Some studies have shown beneficial effects of fiber and changes in the gut microbiota [5, 15], whereas others have not shown any significant change [6, 12, 14]. Results have been dependent on the type of fiber, percentage of fiber, previous diet fed, duration of treatment, health status and methodology used during the analysis. The modern pet food industry uses several fiber sources (mainly by-products derived from the processing of grains, fruits, and vegetables) in the formulation of diets for dogs [15, 16]. There is still a paucity of information regarding the effect of many fiber sources on the composition and activity of the intestinal microbiota of dogs and cats. Similarly, studies have also been published assessing the effect of protein [17–19], and recently with emphasis in obesity [20, 21], and raw meat diets in dogs [8, 22, 23], but more studies are needed to understand this complex interaction under different feeding conditions.

Dysbiosis of the intestinal microbiota has been linked to chronic intestinal inflammation in people, dogs, and cats [24–28]. Chronic intestinal diseases in dogs are often treated by dietary modification, aimed at reducing antigenic stimulation to the intestine [29]. Hydrolyzed diets are composed of low molecular weight (MW) proteins and peptides in order to evade the intestinal immune system [30]. In addition, many commercial veterinary hydrolyzed diets will have other alterations (such as increased polyunsaturated fats) compared to standard veterinary diets [31]. Hydrolyzed diets are associated with beneficial changes in the intestinal microbiota and clinical signs of dogs with chronic enteropathies [32, 33]. However, to our knowledge, there is no published data on how hydrolyzed diets affect the gastrointestinal microbiome in healthy dogs fed different types of diets prior to the change, and in turn, which component of the diet is having the most impact.

The aim of this study was to investigate the effects of dietary modification with a high-protein (all meat) diet, a high-insoluble fiber diet and a hydrolyzed diet on the fecal microbiota of healthy dogs in a cross-over trial.

## Results

### Effect of diet on the relative abundance of bacterial groups

**Fig. 1:**
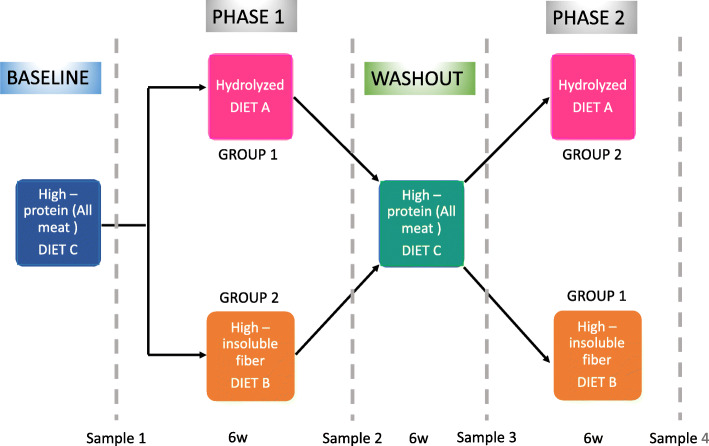
Schematic design of the cross-over over study. Participant dogs switched diets after a washout period. Sample collection (4 time-points). Each stage of the trial consisted in a 6-week period

Dogs were classified into two groups. Group 1 dogs were fed diet sequence ACB, and group 2 dogs were fed diet sequence BCA (A = hydrolyzed diet; B = high-insoluble fiber diet; C = high-protein diet [all meat/carcass, raw diet]), each feeding period lasting for 6 weeks. All dogs were fed with a high-protein diet (diet C [all meat/carcass, raw diet]) at baseline (Fig. 1).

**Fig. 2:**
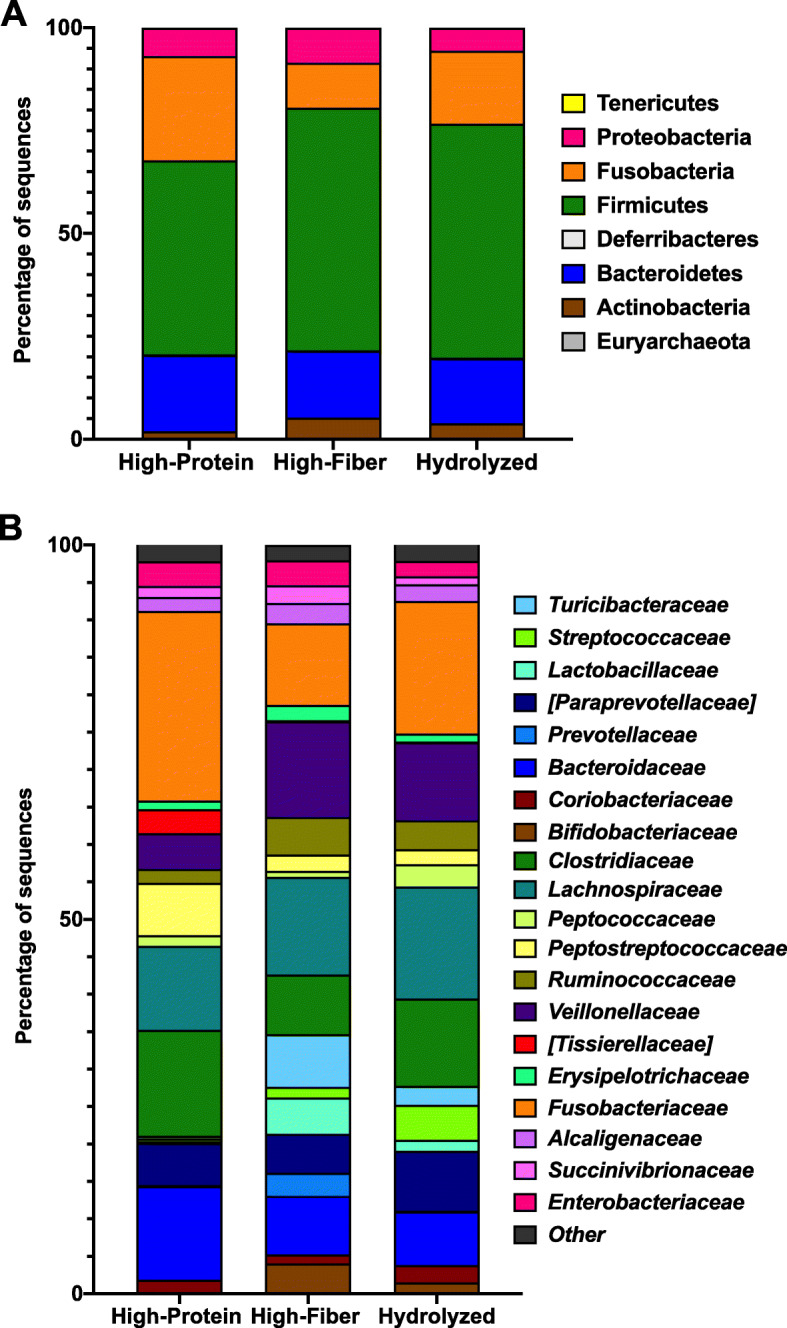
**A** Relative abundance of bacteria at phylum level in the three diets, irrespective of sequence fed. **B** Relative abundance of bacteria at family level with the three diets, irrespective of sequence fed. High-Protein (diet C); Hydrolyzed diet (diet A) and High-insoluble fiber (diet B)

The relative abundance of the different bacteria at phylum and family phylogenetic levels were compared among the different categories of diet. At phylum level, Firmicutes, Bacteroidetes and Fusobacteria were the most populous bacterial phyla found, as previously reported [34]. Firmicutes had a median of 44% [range: 18–91%] with the high-protein diet (diet C), a median of 62% [range: 29–93%] with the high-insoluble fiber diet (diet B) and a median of 55% [range: 30–95%] with the hydrolyzed diet (diet A). For Bacteroidetes, the median was 14% [range: 0.22–50%] for the high-protein diet, 16% [range: 0.44–41%] for the high-insoluble fiber diet and 16% [range: 0.34–51%] for the hydrolyzed diet. Meanwhile, for Fusobacteria the median was 24% [range: 4–72%] for the high-protein diet, 8% [range: 1–45%] for the high-insoluble fiber diet and 17% [range: 2–34%] for the hydrolyzed diet (Fig. 2A). The relative abundances at family level are shown in Fig. 2B.

**Fig. 3:**
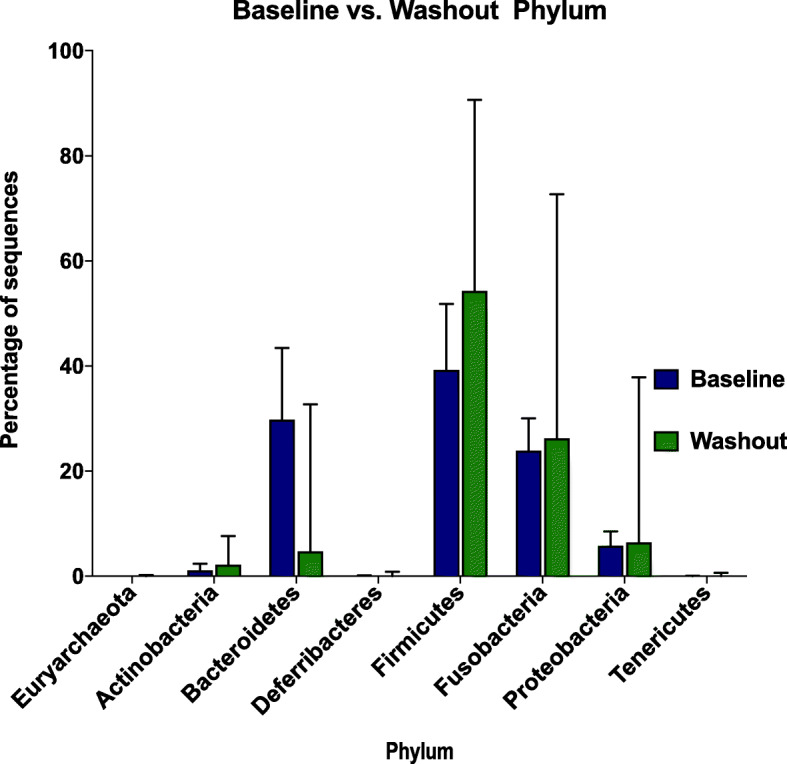
Relative abundance of bacteria at phylum level Baseline versus Washout period. (High-protein diet) (diet C). *N* = 44, *n* = 44 each period. Median with range. N: number of dogs. n: number of samples

Next, we assessed the relative abundance of the different bacterial groups during the baseline and the washout periods (when dogs were being fed diet C- the raw meat/high-protein diet) and found that the relative abundance of some phyla differed between these periods. During baseline, approximately 31% (median) of the sequences corresponded to Bacteroidetes [range: 3–50%], whereas at the end of the washout period, the percentage was 5% [range: 0.22–33%]. For Firmicutes, during baseline the percentage was 37% [range: 18–71%] versus 54% [range: 18–91] during the washout period (Fig. 3). This difference was observed irrespective of the diet sequence for individual dogs.

Analysis of the relative abundance of the different phyla in the hydrolyzed and high-insoluble fiber diets also differed between the ACB and BCA sequences. For example, samples taken from dogs at the end of the 6-week period being fed with the hydrolyzed diet (diet A) showed a relative abundance of Bacteroidetes of 24% (median) [range: 0.71–51] in ACB versus 7% (median) [range: 0.34–29%] in the BCA sequence. However, when the percentages were compared with the preceding diet in each diet sequence; the introduction of the hydrolyzed diet (diet A) did not affect the percentage of Bacteroidetes in any of the diets. In the ACB diet, the percentage of Bacteroidetes ranged between 3 and 50% (median: 23%) at baseline (high-protein, raw meat diet) and for the BCA diet, the percentage ranged between 0.5–33% (median: 8%) at the end of the washout (high-protein, raw diet) period. Thus, changes should be interpreted based on the diet sequence and the preceding microbial profile of each subject, and not independently (See Additional file 1: Fig. S1).

### Dietary effects on gut microbial alpha and beta diversity

Alpha diversity was analyzed using the Shannon index considering the subject, as well as the dietary intervention (time point) and diet sequence. In general, Shannon diversity index was not affected by the change of diet when time and subject were considered; although it was lower in the washout period compared to baseline and slightly higher in BCA diet sequence in comparison to ACB diet sequence. The marginal *R*^2^ is about 0.3, which suggests that the diet and sequence effects together describe about 30% of the variance in Shannon index [35] (See Additional file 2: Table S1).

In response to the diets, we see a large shift in the overall taxonomic composition of the microbiome. Beta diversity principal-coordinate analysis (PCoA) plots constructed using the Bray-Curtis distance showed a clear separation between high-protein (diet C) with the hydrolyzed diet (diet A) and high-insoluble fiber (diet B) diets (See Additional file 3: Fig. S2A; diet sequence ACB and Additional file 4: Fig. S3A; diet sequence BCA). The hydrolyzed and high-insoluble fiber diets used in our study have some similar nutritional characteristics (e.g., total protein, total carbohydrate) compared to the high-protein diet that could explain, in part, the clustering pattern. Permutational multivariate analysis of variance (PERMANOVA) (Adonis) analysis showed that the type of diet explained ~ 20% of the variability in beta- diversity (*R*^2^: 19, *p*: 0.001), whereas diet sequence only explained 1% of the variability (*R*^2^: 1, p: 0.007). When the interaction of these two factors were assessed, diet sequence explained ~ 5% of the variability caused by the type of diet (*R*^2^: 6, *p*: 0.001).

In accordance with the results showed in the relative abundance tables, PERMANOVA (Adonis) analysis using the Bray-Curtis distance identified a significant difference in beta-diversity between the baseline and washout periods (*R*^2^: 25, *p*: 0.001).

Analysis of each group separately, showed that the shifts of the microbiota increased over time when compared to the baseline diet, and was independent of the diet sequence (See Additional file 3: Fig. S2B; diet sequence ACB and Additional file 4: Fig. S3B; diet sequence BCA). In line with the previous clustering pattern, the distance between the hydrolyzed and high-insoluble fiber diet was smaller. The consistency of the community shift argues for a direct effect of the diet as, in the absence of intervention, the dog microbiota has been reported to be stable over time, using 16S rRNA profiling [36].

### Differential dietary effects on gut bacterial phyla and families

**Fig. 4:**
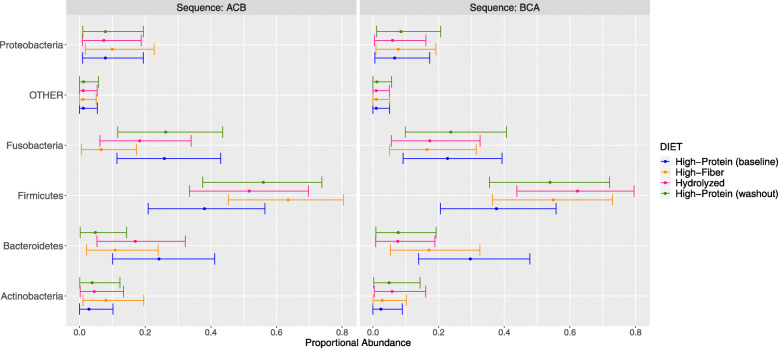
The posterior estimated predictive distribution of relative abundances at phylum level in ACB and BCA sequence. Points are the posterior population mean. The bars are the 89% prediction intervals from the posterior distribution. Inter-subject variation is not included. Baseline [high-protein] (diet C), High-insoluble Fiber (diet B), Hydrolyzed (diet A) and Washout [high-protein] (diet C)

A Dirichlet regression model was performed to compare the microbial differential abundance in each diet sequence considering the variation between dogs and the diets. At phylum level, the high-protein diet (diet C) was enriched with Fusobacteria, for the ACB diet sequence, whereas the high insoluble-fiber (diet B) and hydrolyzed diet (diet A) induced an enrichment in the Firmicutes phylum. Firmicutes was also enriched in the washout period but not during the baseline, whereas Bacteroidetes was enriched only at baseline but not during the washout period. In addition, Actinobacteria was enriched in the high-insoluble fiber diet but only in the ACB sequence (Fig. 4).

**Fig. 5:**
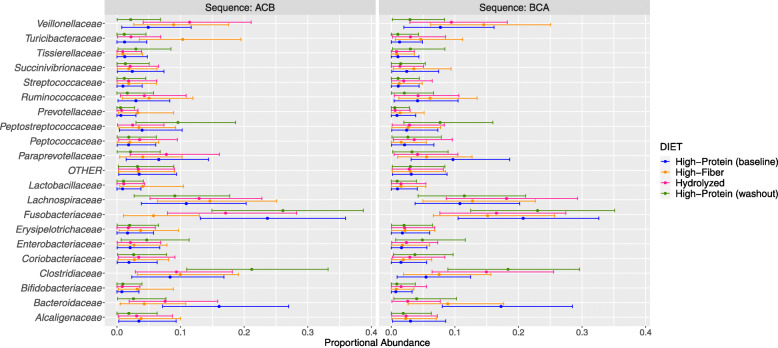
The posterior estimated predictive distribution of relative abundances at family level in ACB and BCA sequence. Top of the 20 most abundant families. Points are the posterior population mean. The bars are the 89% prediction intervals from the posterior distribution. Inter-subject variation is not included. Baseline [high-protein] (diet C), High-insoluble Fiber (diet B), Hydrolyzed (diet A) and Washout [high-protein] (diet C)

At family level, the 20 most abundant families were assessed. These results were also dependent on the diet sequence, suggesting that the outcome of dietary intervention is influenced by the previous dietary history and the baseline microbiome of the individual. For example, *Turicibacteraceae*, *Lactobacillaceae*, *Bifidobacteriaceae* and *Erysipelotrichaceae* were more abundant in the high-insoluble fiber samples, but only in the ACB diet sequence. *Peptostreptococcaceae* and *Clostridiaceae* were more abundant in the high-protein (diet C) samples only during the washout period, whereas *Bacteroidaceae* was more abundant only at baseline, and *Fusobacteriaceae* was more abundant in both periods, regardless of diet sequence. For the hydrolyzed diet, only *Veillonellaceae* was more abundant in comparison with the other diets, but only during ACB diet sequence (Fig. 5). This family was also more abundant in diet B. *Veillonellaceae* has been positively correlated with fiber intake [37].

At the genus level, the ratio of *Prevotella* to *Bacteroides* has also been found to be important in the human gut microbiome; it changes in response to diet, with higher *Prevotella* relative abundance being observed with high carbohydrate diets, while higher relative abundance of *Bacteroides* has been associated with a high-protein diet [38]. In our study, we observed that the ratio of *Prevotella* to *Bacteroides* was higher in the hydrolyzed and high-insoluble fiber diets compared to the high-protein diet (See Additional file 5: Fig. S4).

### Functional changes in the gut microbiota

Phylogenetic investigation of communities by reconstruction of unobserved states (PICRUSt) was performed on the 16S rRNA gene gut bacterial composition data to predict Kyoto Encyclopedia of Genes and Genomes (KEGG) orthologs (KOs) and pathways [39]. All predicted KO pathways at L2 level were subjected to a linear mixed-effects model considering the type of diet and the diet sequence as predictors of the effect of each functional pathway. However, there were no clear effects of diet type or sequence on the predicted metagenome functional content.

## Discussion

Several studies have been conducted on the effect of diet on the gut microbiota in humans and rodents, and more studies are emerging in dogs. However, the gut microbiota-diet relationship is complex and challenging to characterize as many factors may influence the results [2]. In our study, all dogs were receiving the same baseline diet, were the same breed, similar age, similar body condition and lived in the same environment, which served to eliminate many confounders that could influence the results. In general, we observed that the selected diets had a substantial residual impact on the fecal communities of all dogs, and results were dependent on the composition of the gut microbiota at the start of the intervention.

Analysis of the alpha diversity between the different categories of diet, showed that overall, the high-insoluble fiber diet and hydrolyzed diet have a higher Shannon diversity in comparison with the raw meat, high-protein diet. However, when the analysis was done per subject, the difference in Shannon diversity was minimal between diets.

Studies investigating the direct impact of protein on gut microbiota composition and functionality have shown that protein quality and source are as important as total amounts in people [2], but less so in dogs [40]. Analysis of the gut microbiota showed that diet C (raw all-meat/high-protein diet) in our study was characterized by an overrepresentation of bacteria belonging to the Fusobacteria phylum. This contrasts with a previous study made in obese and lean dogs with high-protein dietary intervention, where the changes in Fusobacteria were relatively small, although the levels of protein differed between studies (49.38% vs. 19.8% in ours) and was of a shorter duration [20]. Another study where the dry commercial diet was changed to minced raw beef, also showed minimal changes in the Fusobacteria content [17]. High levels of *Fusobacterium sp.* have been observed in multiple carnivore species [3, 41, 42]. In humans, increased Fusobacterium levels are seen in people consuming a diet high in red and processed meats and are associated with an increased risk of development of colorectal cancer [43, 44]. In one study of dogs fed a red meat diet for 9 weeks, an increased Fusobacterium abundance (~ 15%) was present at 6 weeks, suggesting these changes may take time to develop [19]. Diet C in our study consisted mainly of horse carcass, which is a vastly greater percentage of protein in diet than most commercial diets (prescription or supermarket brand). Digestibility of scrap meat may be lower than for high quality protein due to the high amount of connective tissue, and digestibility of macronutrients may also influence the colonic microbiome [45]. Additionally, most commercial dog food does not contain horse protein, which may be biologically different than other sources of protein [46, 47].

At lower phylogenetic levels, an overrepresentation of members of the families *Clostridiaceae* and *Peptostreptococcaceae* were also found in the samples from dogs fed diet C. *Clostridium* is important for lysine and proline utilization by the host, via fermentation in the colon; while Peptostreptococci drives tryptophan and glutamate catabolism [48]. In people, an exclusively meat-based diet is frequently associated with high levels of bile-tolerant bacterial species like *Bacteroides* and low levels of *Prevotella* [38]. Of interest, in our study, *Clostridiaceae* and *Peptostreptococcaceae* were only enriched in the washout period, whereas the *Bacteroidaceae* family was enriched during baseline, emphasizing the effects of previous diet on the microbiota profile.

Studies that have evaluated the impact of low-fiber/high-protein meat-based raw diets in the gut microbiome of healthy dogs have shown an overall decrease in the abundance of Firmicutes, including genera *Peptostreptococcus* and *Faecalibacterium*; and in *Bacteroides* and *Prevotella* (phylum Bacteroidetes) [22, 23, 49]. Conversely, other bacterial taxa were found to increase in abundance, including Proteobacteria and Fusobacteria (genus *Fusobacterium*) [22, 23], and two genera from phylum Firmicutes (*Lactobacillus* and *Clostridium*) [22, 49].

Although previous studies have identified increased levels of *Enterobacteriaceae* in dogs fed raw diets, we did not see enrichment of this bacterial group during this dietary intervention [49].

The high-insoluble fiber diet (diet B) used in this study contains 25.5% (dry matter [DM]) insoluble fiber and 1.9% DM soluble fiber; the total dietary fiber is therefore 27.6% DM and crude fiber 16.4% DM. This type of diet is used for conditions such as weight loss, diabetes mellitus, chronic pancreatitis and historically for conditions like colitis. Most standard canine diets fed for maintenance in adults contain crude fiber around 1.5–5% DM. Diet B in our study induced an enrichment in bacteria belonging to the Firmicutes and Actinobacteria phyla. However, at family level, *Prevotellaceae* (belonging to the Bacteroidetes phylum) was also enriched. This agrees with human studies, where it has been found that increased levels of *Prevotella* are associated with a plant-based diet rich in fiber, simple sugars, and plant-derived compounds, as they harbor genes for cellulose and xylan hydrolysis [38, 50].

Hydrolyzed diets are used frequently in dogs for the treatment of putative food allergies and chronic enteropathy [30, 33, 51]. The main difference between a commercial dry diet designed for healthy dogs and a hydrolyzed diet is that the latter is composed of smaller chains of protein or single amino acids that decreases the probability of an immune response to protein dietary components [30]. The diet used (diet A) is based on hydrolyzed poultry, and although has lower fiber content and higher fat content than diet B, it is similar in overall macronutrient composition to commercial maintenance diets. Evaluation of the effect of the hydrolyzed diet did not show overrepresentation of any member at the phylum and family phylogenetic levels, in comparison with the other two diets. Potentially, dietary impact of hydrolyzed diets on the gut microbiota could be at functional level and not necessarily at taxonomic levels. Although we used PICRUSt to predict community functional capabilities [39], we could not determine if any functional changes had actually occurred. Further studies assessing function (metabolomics), and strains (metagenomics) could help us to elucidate the relevance and the role of these microbiota changes in the gut. It is interesting that these changes were different and independent from the high fiber diet, which suggests a different impact on the microbiota.

Recent studies evaluating the effect of a hydrolyzed diet on the gut microbiome in healthy dogs and in dogs with food-responsive enteropathy showed that the impact of the diet was minimal in the microbial composition as well as in the metabolome [40, 52]. In these studies, dogs were fed with commercial maintenance diets before the introduction of the new diet, whereas in our study the baseline diet was meat-based, which could potentially influence the results. Also, the percentage of fat differs among hydrolyzed diets, with our diet being slighter higher in fat percentage.

We also saw that the magnitude and nature of the changes induced by the high-insoluble fiber and hydrolyzed diets varied according to the diet sequence. The initial bacterial composition, the fact that bacteria form a metabolic network and cross-feed each other and that there is significant heterogeneity within bacterial species in their ability to digest different types of fiber [2, 53] add complexity to the diet-microbiota interaction. In people, particularly in the case of fiber, it has been shown that an individual’s baseline microbiota harbors predictive potential with regards to the effect of dietary constituents on the host [54]. Also, we should take into consideration that the proportion of one macronutrient to the total energy intake inherently influences the contribution from other macronutrients. Thus, the effect of a change in one macronutrient on the fecal microbiota is a result of the combinatory effect of all the macronutrients [55].

In our study, we observed that the ratio of *Prevotella* to *Bacteroides* was higher with the hydrolyzed and high-insoluble fiber diets compared to the high-protein diet. In accordance with this, it has been reported that a high fiber diet correlates with a polysaccharide-utilizing microbiota with lower protein fermentation products and fewer Bacteroides and Clostridia [56, 57]. However, when we analyzed the families using the Dirichlet model, we observed that *Prevotellaceae* was only higher in the high-insoluble fiber diet and only in the ACB sequence, whereas members of the *Bacteroidaceae* family were higher in the high-protein diet but only during the baseline period.

Finally, assessment of the gut microbiota during the washout period showed that the gut microbiota of dogs did not revert to their original phylogenetic structure after 6 weeks. Previous studies in dogs have reported adaptation periods varying from 10 days to 4 weeks [5, 6, 8, 23, 58]. In our study, although the washout period was longer than previously reported, changes in the composition of the gut microbiota persisted over time. This was evidenced by sequence and diet effects and by differential results in bacterial abundance between baseline and washout periods. These changes could be permanent, or there is a possibility that more time is required to return to baseline levels. The intestinal microbiota is reported to be resistant to most environmental influences, returning rapidly to its pre-treatment state, particularly for short-term interventions, suggesting in our dogs that only diet was responsible [4]. Furthermore, studies have shown that long term changes to dietary habits may be required to achieve permanent changes in the gut community structure [38]. However, this can depend on the magnitude and duration of the change being study [4, 59].

The limitations of this study were the presence of only one breed, age (although they were evenly distributed in both groups), and that potentially the manufacturing process of the commercial diets themselves could have influenced the gut microbiota. The alternate day feeding pattern of diet C could also have influenced microbiome composition independently of the protein/digestibility [60, 61]. Furthermore, day to day variations in microbiota occurs and, in our study, feces were only collected at a set time point [36]. Pooling samples over a collection period of several days may have been more beneficial to average out day-to-day variability but would have added more complexity to the analysis.

In addition, evaluation of microbial composition together with functional analysis (metabolomics, transcriptomics) would offer a better insight into the total effect of diets [53]. Different microbiomes have different potentials for producing certain metabolites, depending on the metabolic capabilities and metabolic interactions within the population. The fact that a bacterium harbors a gene does not imply that the gene is expressed. In the presence of different energy sources, bacteria may express genes to produce one, a group or several of these enzymes, depending on the environmental context [53]. Future studies could combine several approaches to elucidate the influence of the diet-microbiota interaction on host biology.

## Conclusion

This study demonstrated that that dietary protein and fiber ratios can impact the gut microbial composition. Alterations on the microbiota structure are dependent on the bacterial composition present at the time of intervention, as results were quite susceptible to study design, evidenced by sequence and diet effects. Further functional studies are required for a better understanding of the ways the dietary-microbiome crosstalk interacts with the host. This will allow, in the future, the implementation of targeted and effective dietary interventions for the alleviation of microbiome-associated diseases.

## Methods

### Study dogs

Fifty healthy foxhound dogs (all lean body weight, body condition score [BCS] range 3–5/9 Purina body condition score system) were enrolled in the study [62]. All dogs had up to date vaccination status and no signs of gastrointestinal disease or medication within the previous 3 months. All dogs enrolled underwent a full physical examination, complete blood count and biochemical profile. They were dewormed with praziquantel 200 mg, pyrantel 560 mg and oxantel embonate 2180 mg (Paratak™ Plus) on two separate occasions 12 weeks apart prior to commencement of the trial.

The dogs were normally fed a high-protein (all meat/carcass) diet every second day. For the study, the dogs were kept in two groups of 25 each, physically separated during the study. The two groups had access to communal drinking water in their allocated yards, obtained from the same source, and were located close to each other, but had no contact with animals from the other group for the duration of the study. All environmental factors were the same for both groups (shelter, bedding, exercise area etc.).

Group 1 contained 10 males and 13 females with a mean age of 5.3 ± 2.5 (SD). Group 2 contained 16 males and 5 females with a mean age of 3.7 ± 2. Two dogs in group 1 were excluded during the feeding trial period, one due to illness, the other as it refused to eat the trial food. Four dogs were excluded from group 2 during the feeding trial period: three due to antibiotic use and one due to inadequate fecal sample collection at one time point.

Each group was randomly assigned to be fed one of the two experimental diets: diet A (hydrolyzed) (Hill’s® Prescription Diet® z/d® Canine) or diet B (high-insoluble fiber) (Hill’s™ Prescription Diet™ w/d™) diet daily for 6 weeks (Phase 1). Following this, there was a washout phase of 6 weeks when dogs returned to their normal high-protein diet (all meat/carcass, raw diet) (diet C) fed alternate days. The groups were then crossed over to receive the alternative diet for 6 weeks (Phase 2). Dogs were fed to maintain body weight once daily and had free access to water (Fig. 1).

### Samples

Individual fecal samples were obtained at 4 time points: baseline, after 6 weeks of the first diet (diet A or B), after 6 weeks of washout (on baseline diet) and after 6 weeks on the second diet (crossing over to A or B). A total of 176 samples were collected.

### Diet composition and analysis

The main source of protein in diet A was hydrolyzed chicken liver, whereas for diet B the main source was soybean meal. The main source of carbohydrate (CHO) in diet A was corn starch and cellulose and for diet B was soybean meal. Regarding fiber, diet A was mainly composed of powdered cellulose and diet B of soybean meal and dried beet pulp. A detailed list of ingredients of the commercial diets can be found in Additional file 6: Table S2.

The following analysis of diets A and B were obtained directly from the manufacturer (as diet is not the same as currently produced) (Table 1). The content of diet C (horse meat carcass- bones, muscles, ligaments but no organs) was analyzed using online diet composition and published references of horse meat composition for protein, fat, CHO (http://www.foodnutritiontable.com/nutritions/nutrient/?id=132. Page accessed April 2020) [46, 47].

**Table 1:** Nutrient composition of the different diets

Diet	Fat % Dry matter (DM)	Fat g/100 kcal ME*	Protein % DM	Protein g/100 kcal ME*	Crude fiber % DM	Crude fiber g/100 kcal ME*	CHO (NFE) % DM	CHO (NFE) g/100 kcal ME*	Ash % DM	Kcal/100 g
A	14.4	4	19	5.3	2.9	0.8	58.4	16.2	5.3	360
B	8.7	2.91	19.2	6.4	16.4	5.5	50.8	16.99	5.0	299
C	6.63	6.14	21.1	19.54	0	0	0	0	0.5	108

A: Hydrolyzed, B: High-insoluble fiber, and C: Horse carcass.^*^As fed. *ME* Metabolizable energy, *DM* Dry matter, *NFE* Nitrogen-free extract

### Fecal DNA extraction

All samples were collected upon voiding without contacting the environment (to avoid transfer of genetic material) or via rectal collection. Samples were refrigerated at 4 °C until transport to the laboratory, which was completed within 48 h of sample collection. Samples were then frozen and stored at − 80 °C until processing.

Fecal DNA was extracted using the Power soil DNA isolation kit (MoBio® laboratories Catalog No. 12888–100); 250 mg of feces were processed using the protocol for DNA isolation, detailed in the manufacturer’s instructions, with some modifications. Briefly, the fecal pellet was added to a glass bead tube (0.1 mm) and 750 μL of bead solution and 60 μL of C1 solution were added. Then, samples were incubated at 94 °C for 10 min. Afterwards, tubes were placed in the PowerLyzer® 24 and were run at 3000 rpm for 45 s. Subsequent steps were done as indicated by the manufacturer. Extracted DNA was eluted from the spin column in 100 μL of C6 solution from Mobio® (10 mM tris-Cl pH 8.0–8.5). Extracted DNA was quantified and checked for purity, based on UV absorption ratios 260:280 nm and 260:230 nm, on a ND1000 spectrometer (NanoDrop™ 2000/2000c). Samples with highly aberrant absorption ratios were re-extracted.

### Bacterial 16S rRNA gene analysis

Illumina sequencing of the V4 region of the bacterial 16S rRNA genes was performed using primers 515F (5′-GTGCCAGCMGCCGCGGTAA-3′) to 806R (5′- GGACTACVSGGGTATCTAAT-3′). Raw data was analyzed using the open-source software package Quantitative Insights into Microbial Ecology (QIIME) [63]. Version 1 (QIIME1, release 1.9.0). The sequence data was demultiplexed, and then quality filtered using the default settings for QIIME. Chimeras were detected and filtered from the reads using USEARCH [64] against the 97% clustered representative sequences from the Greengenes v 13.8 database [65]. The remaining sequences were clustered into Operational Taxonomic Units (OTUs) by using an open reference approach in QIIME [63].

From 176 samples, a total of 11.650.924 high-quality sequences were obtained, with the number of reads ranging from 12.391 to 165.430 per sample (median 60.448; mean 65.824.429; standard deviation (SD) 29.494.371). Samples were rarefied at 12.390 sequences per sample for even depth of analysis.

Rarefaction plots were used to visualize adequacy of depth in the sequencing data. Measurements of Alpha (α) – diversity and beta (ß)-diversity were done using QIIME1 and Phyloseq package (version1.18.1) [66]. Beta-diversity was assessed using the Bray–Curtis dissimilarity metrics.

Phylogenetic investigation of communities by reconstruction of unobserved states (PICRUSt) [39] was used to predict functional gene content based on 16S rRNA gene data present in the Greengenes database and the KEGG database, using the “*predict_metagenomes.py*” command in PICRUSt (v1.0.0) (http://picrust.github.io/picrust/).

### Statistical analyses

Shannon index (alpha diversity) was defined as the response in a linear mixed model, which included a subject-level random intercept. Fixed effects were the diet, sequence, and interaction of diet and sequence. The model was defined using the ‘lme4’ package in R [67]. Informativeness of the model was assessed using the marginal and conditional coefficients of determination as implemented in the ‘muMin’ package [35, 68].

The same model was used for predictive functional analysis. The L2 level was chosen, and the pathway was defined as the response in the linear mixed model which included a subject-level random intercept. The responses were log-transformed for the analysis. The package ‘emmeans’ from R, was used for post-hoc comparisons among diets and sequences and for estimating marginal means and their 95% confidence intervals [69].

Associations between the diet, and sequence, and the relative abundance of phyla and families were assessed using a hierarchical Dirichlet regression model with the logit link function [70]. The response variables were the proportional abundances of 20 families, where *Bifidobacteriaceae* was the reference level, or the proportional abundances of 5 phyla, where *Actinobacteria* was the reference level. The sum-to-one compositional constraint in the family abundances was handled by the Dirichlet response distribution. A handful of zeros in the original abundance data, disallowed in the Dirichlet distribution, were arbitrarily adjusted, and an ‘OTHER’ category was generated to capture the proportion remaining (satisfying the sum-to-one constraint). Between-dog variability in the intercept for each bacterial family was estimated to accommodate the repeated-measures structure. The model was implemented in R [71] using the ‘brms’ package [72]. The MCMC (Markov chain Monte Carlo) sampling used 4 chains of 10,000 iterations. Chain convergence was assessed visually and by the potential scale reduction statistic *R^*. Priors for the regression coefficients were set as N(0,5), intended to be minimally informative. Due to interpretational difficulty associated with the interdependence of the parameter estimates across families and phyla, the final model was assessed using the posterior predicted abundances across groups and their 89% prediction intervals (considering the residual variation) (‘predicted’ interval plots) and are represented in Fig. 4 (phylum) and 5 (family). The posterior estimated population mean of relative abundances with 89% credible intervals (‘fitted’ interval plots) can be found in Additional file 7: Fig. 5 (phylum) and Additional file 8: Fig. 6 (family). Values of the ‘predicted’ and ‘fitted’ interval plots at phylum and family level are also reported (See Additional file 9: Table S3 [phylum] and Additional file 10: Table S4 [family]).

## Supplementary Information

**Additional file 1: Figure S1.** Relative abundance of bacteria before (high-protein) and after the introduction of the new diet. A: Hydrolyzed diet (diet A) B: High-insoluble fiber diet (diet B). Top 5 most abundant phyla. ^a^: Baseline [high-protein] (diet C) ^b^: Washout [high-protein] (diet C). Median with range.

**Additional file 2: Table S1.** Estimates of the Linear mixed model for Shannon Index.

**Additional file 3: Figure S2.** A: PCoA of Bray-Curtis dissimilarity index on diet sequence ACB and distributions of samples along the PC1 by diet. The percentage of variation explained by the principal coordinates (PC1 and PC2) is indicated on the axes. B: Bray-Curtis distance boxplots of the differences in relative abundance between the baseline and the post-treatment sample from the same dog, in diet sequence ACB. Baseline [high-protein] (diet C), High-Fiber (diet B), Hydrolyzed (diet A) and Washout [high-protein] (diet C).

**Additional file 4: Figure S3.** A: PCoA of Bray-Curtis dissimilarity index on diet sequence BCA and distributions of samples along the PC1 by diet. The percentage of variation explained by the principal coordinates (PC1 and PC2) is indicated on the axes. B: Bray-Curtis distance boxplots of the differences in relative abundance between the baseline and the post-treatment sample from the same dog, in diet sequence BCA Baseline [high-protein] (diet C), High-Fiber (diet B), Hydrolyzed (diet A) and Washout [high-protein] (diet C).

**Additional file 5: Figure S4.** A: *Prevotella* and B: *Bacteroides* relative abundances as a function of the diet. C: Ratios between the two genera in the different categories of diet.

**Additional file 6: Table S2.** List of ingredients commercial diets.

**Additional file 7: Figure S5.** The posterior estimated population mean of relative abundances at phylum level in diet sequence ACB and BCA. Points are the posterior population mean. The bars are the 89% credible intervals. Inter-subject variation is not included. Baseline [high-protein] (diet C), High-Fiber (diet B), Hydrolyzed (diet A) and Washout [high-protein] (diet C).

**Additional file 8: Figure S6.** The posterior estimated population mean of relative abundances at family level in diet sequence ACB and BCA. Top of the 20 most abundant families. Points are the posterior population mean. The bars are the 89% credible intervals. Inter-subject variation is not included. Baseline [high-protein] (diet C), High-Fiber (diet B), Hydrolyzed (diet A) and Washout [high-protein] (diet C).

**Additional file 9: Table S3.** Hierarchical Dirichlet regression model values of the (A) ‘predicted’ and (B) ‘fitted’ interval plots at phylum level.

**Additional file 10: Table S4.** Hierarchical Dirichlet regression model values of the (A) ‘predicted’ and (B) ‘fitted’ interval plots at family level.

## Data Availability

Sequence data generated during this study are available through National center for biotechnology information (NCBI)‘s Sequence Read Archive (SRA) under the BioProject number PRJNA641482. Codes for analysis of the 16S rRNA gene sequencing data, for generation of the figures and for the statistics can be found in the following Github repository: https://github.com/Lina-Maria/Cross-over-diet-study. All other data is included in this published article and its supplementary information files.

## Abbreviations

BCS: Body condition score
CHO: Carbohydrate
DM: Dry matter
KEGG: Kyoto Encyclopedia of Genes and Genomes
MW: Molecular weight
MCMC: Markov chain Monte Carlo
NCBI: National center for biotechnology information
NFE: Nitrogen-free extract
NHMRC: National Health and Medical Research Council
OTU: Operational taxonomic unit
PC1: Principal-coordinate 1
PC2: Principal-coordinate 2
PCoA: Principal-coordinate analysis
PERMANOVA: Permutational multivariate analysis of variance
PICRUSt: Phylogenetic Investigation of Communities by Reconstruction of Unobserved States
QIIME: Quantitative Insights into Microbial Ecology
SD: Standard deviation
SRA: Sequence Read Archive
