# Supplementary figures and images for: Effect of sequentially fed high protein, hydrolyzed protein, and high fiber diets on the fecal microbiota of healthy dogs: a cross-over study
Source: Anim Microbiome. 2021 Jun 11;3:42. doi: 10.1186/s42523-021-00101-8 (PMC8194187; doi:10.1186/s42523-021-00101-8)

**A**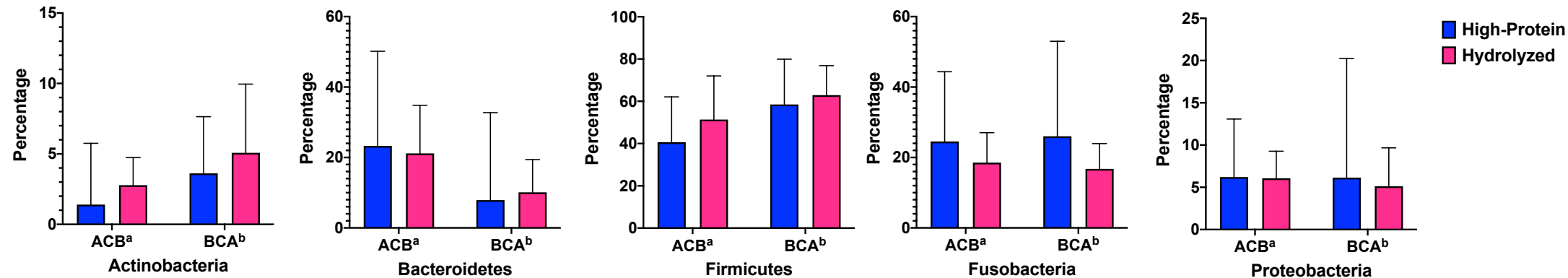**B**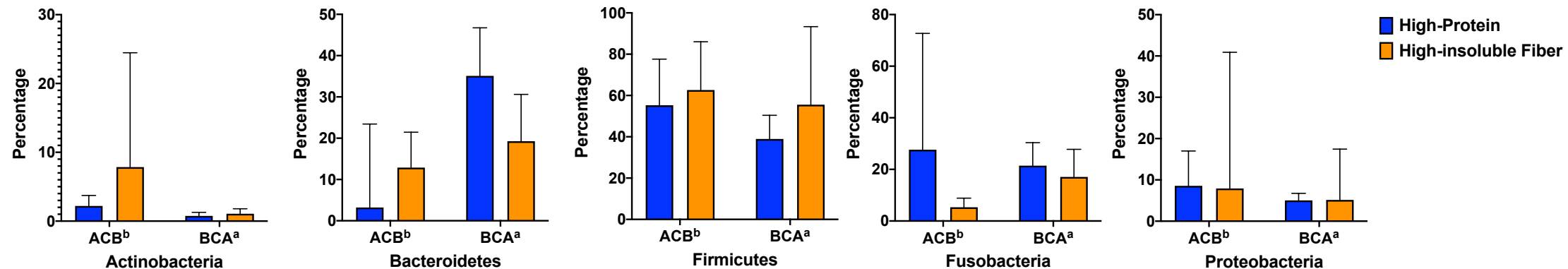

Supplement: Supplementary file 1 — Additional file 1: Figure S1. Relative abundance of bacteria before (high-protein) and after the introduction of the new diet. A: Hydrolyzed diet (diet A) B: High-insoluble fiber diet (diet B). Top 5 most abundant phyla. a: Baseline [high-protein] (diet C) b: Washout [high-protein] (diet C). Median with range. [file 42523_2021_101_MOESM1_ESM.pdf]

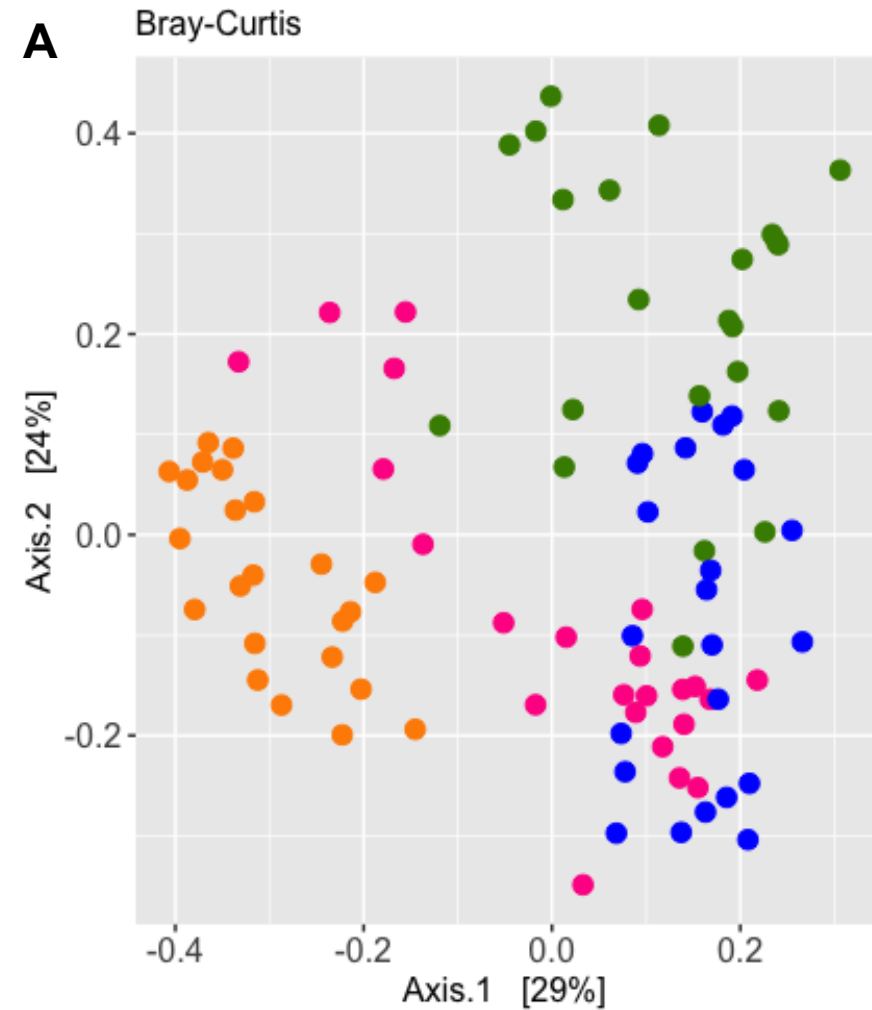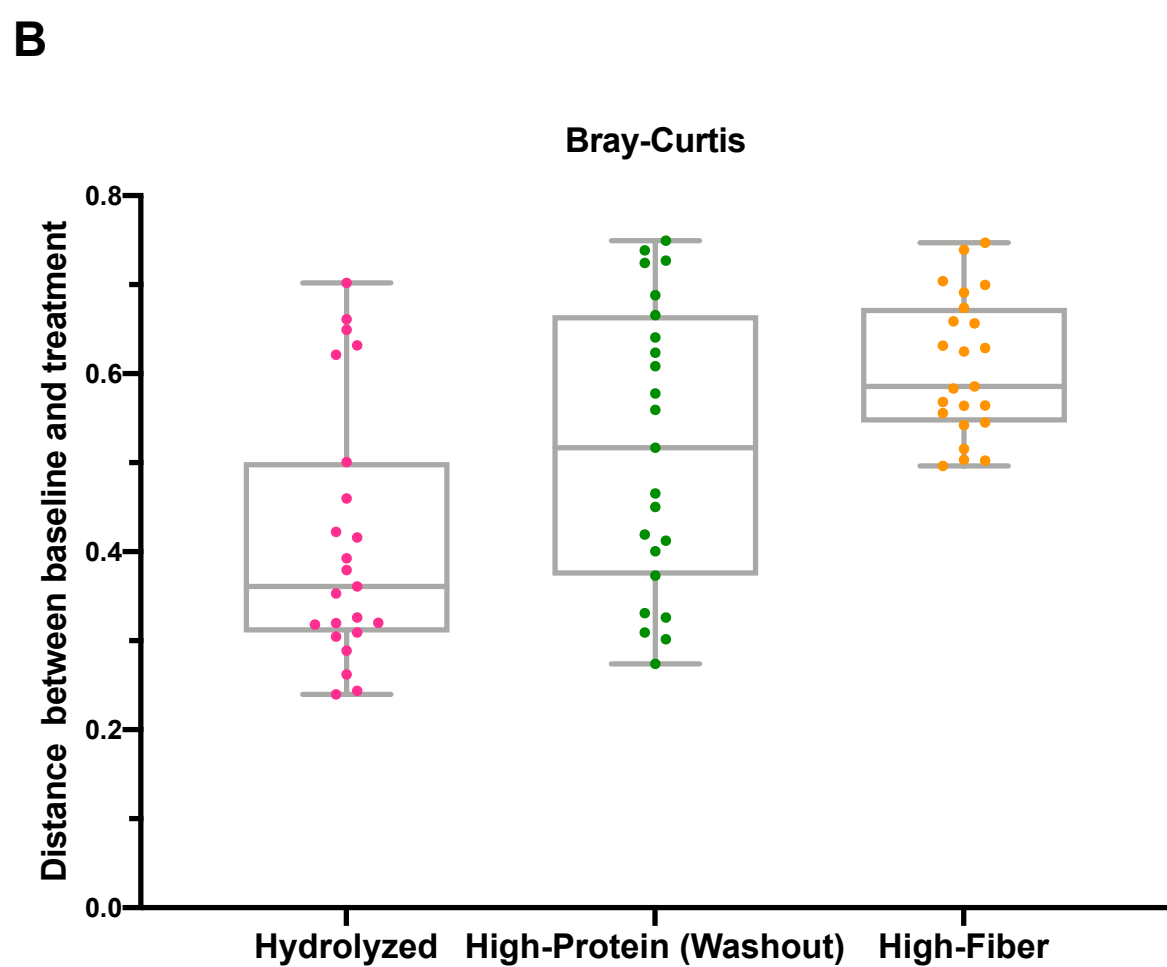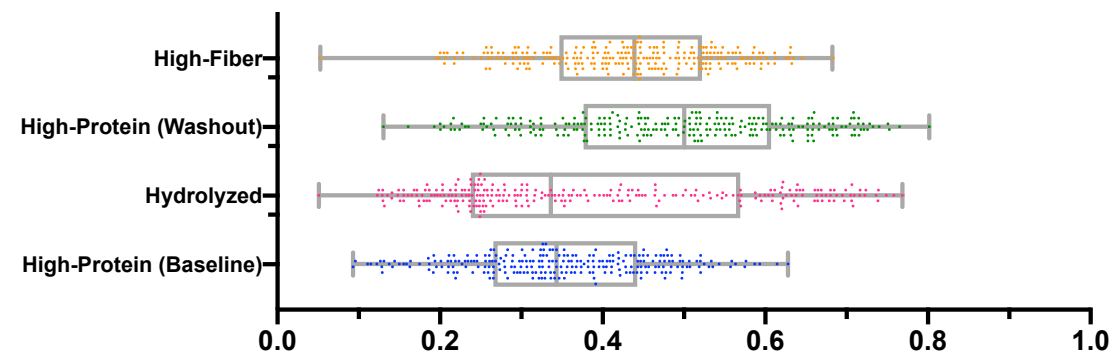

Supplement: Supplementary file 3 — Additional file 3: Figure S2. A: PCoA of Bray-Curtis dissimilarity index on diet sequence ACB and distributions of samples along the PC1 by diet. The percentage of variation explained by the principal coordinates (PC1 and PC2) is indicated on the axes. B: Bray-Curtis distance boxplots of the differences in relative abundance between the baseline and the post-treatment sample from the same dog, in diet sequence ACB. Baseline [high-protein] (diet C), High-Fiber (diet B), Hydrolyzed (diet A) and Washout [high-protein] (diet C). [file 42523_2021_101_MOESM3_ESM.pdf]

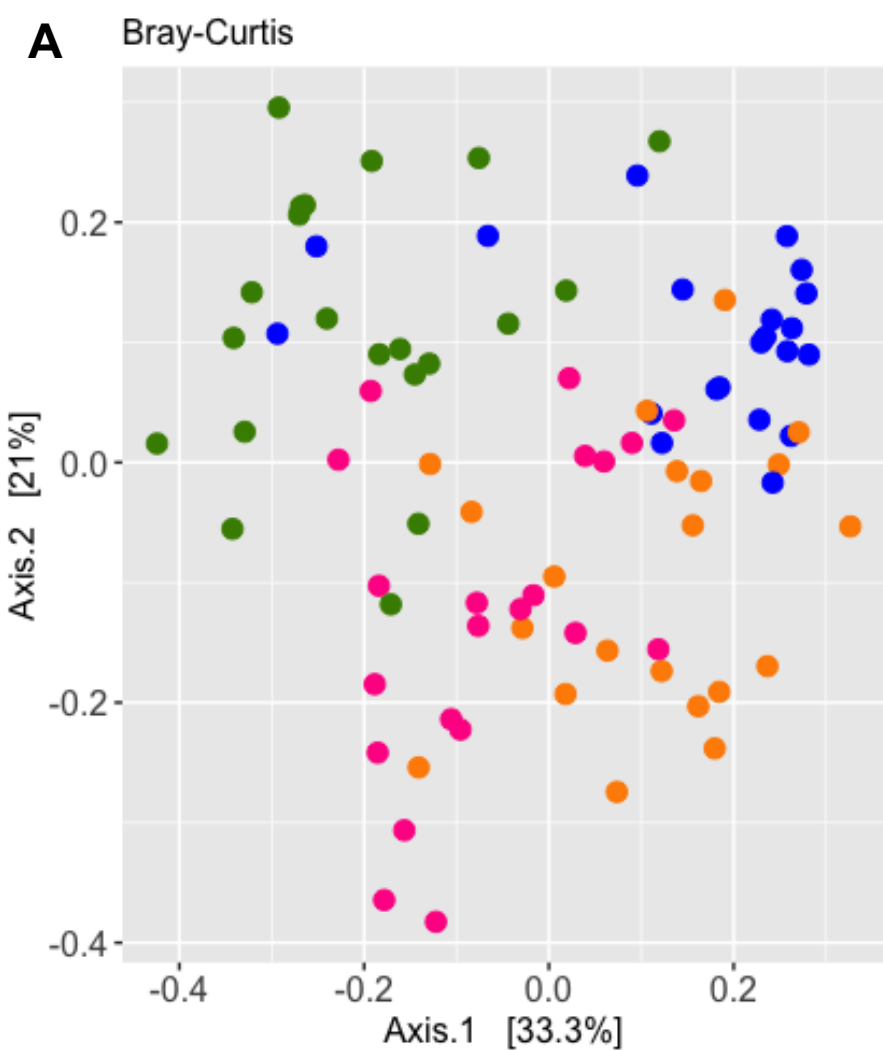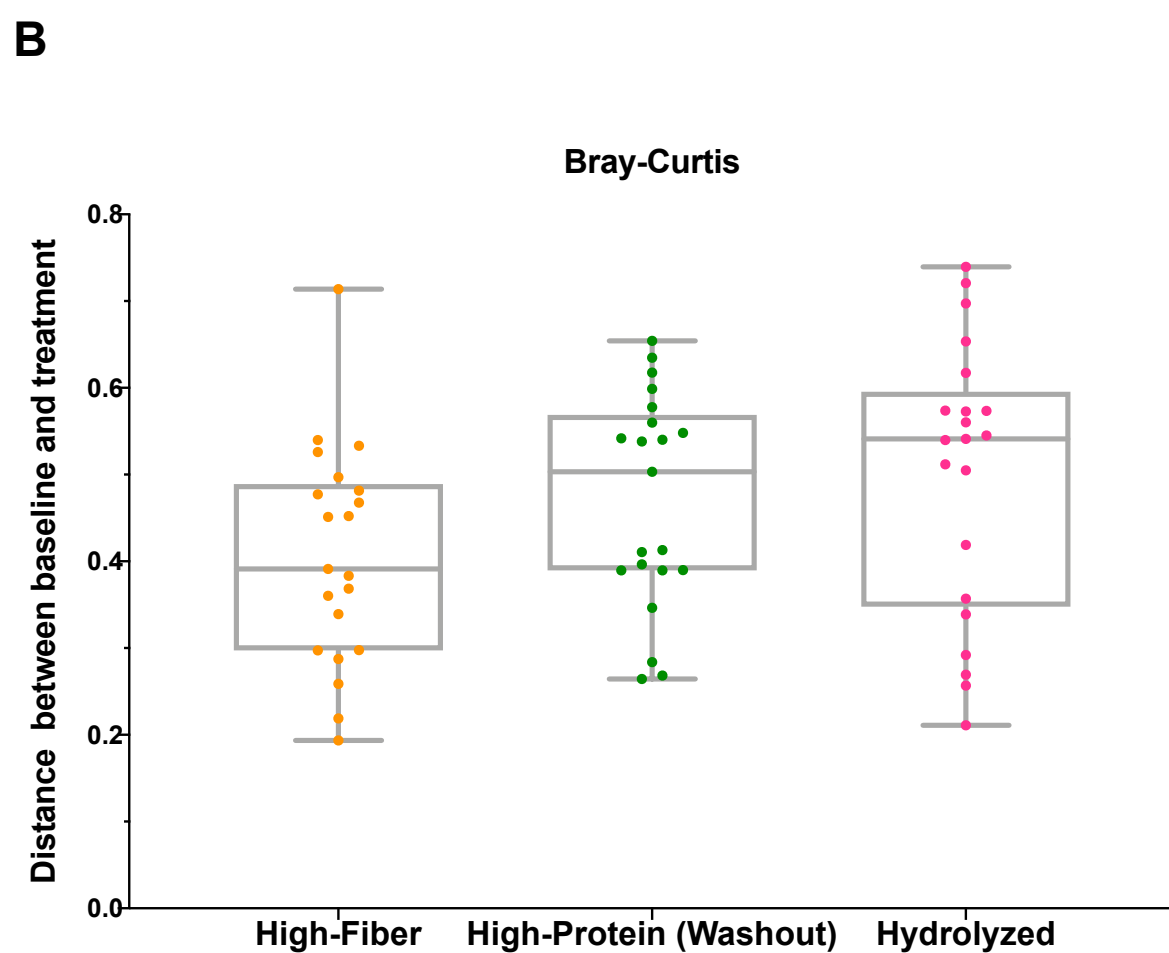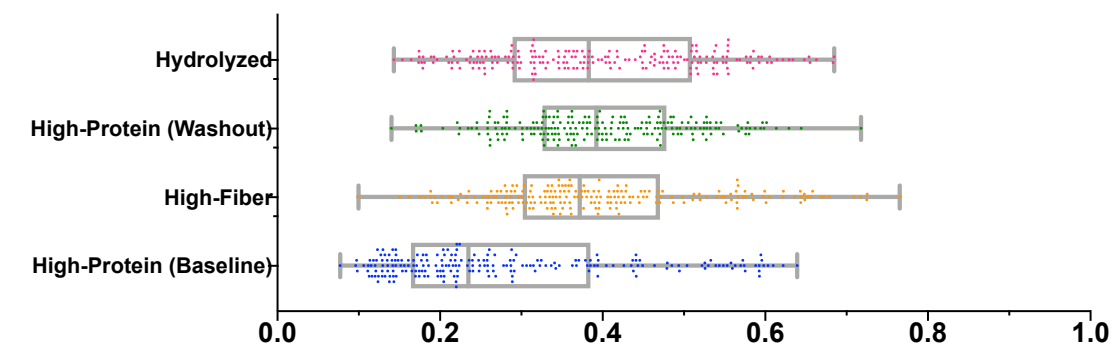

Supplement: Supplementary file 4 — Additional file 4: Figure S3. A: PCoA of Bray-Curtis dissimilarity index on diet sequence BCA and distributions of samples along the PC1 by diet. The percentage of variation explained by the principal coordinates (PC1 and PC2) is indicated on the axes. B: Bray-Curtis distance boxplots of the differences in relative abundance between the baseline and the post-treatment sample from the same dog, in diet sequence BCA Baseline [high-protein] (diet C), High-Fiber (diet B), Hydrolyzed (diet A) and Washout [high-protein] (diet C). [file 42523_2021_101_MOESM4_ESM.pdf]

**A**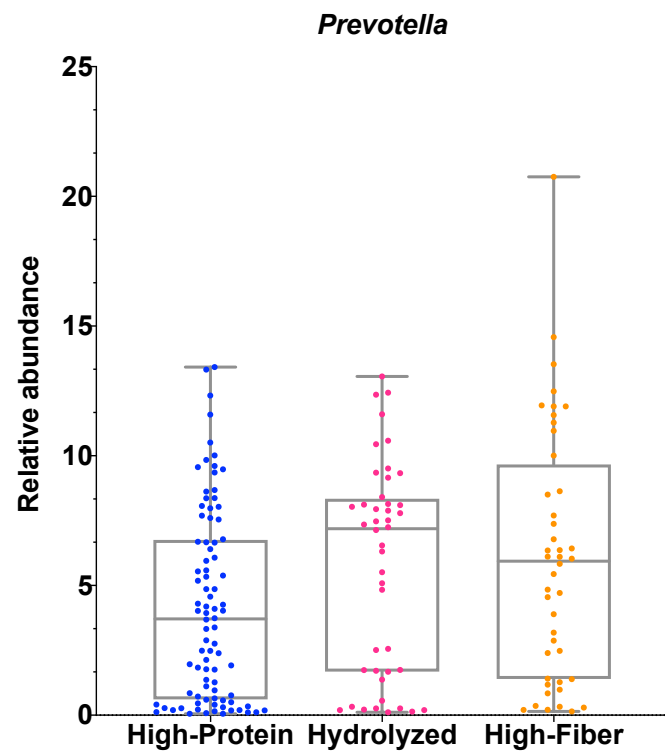**B**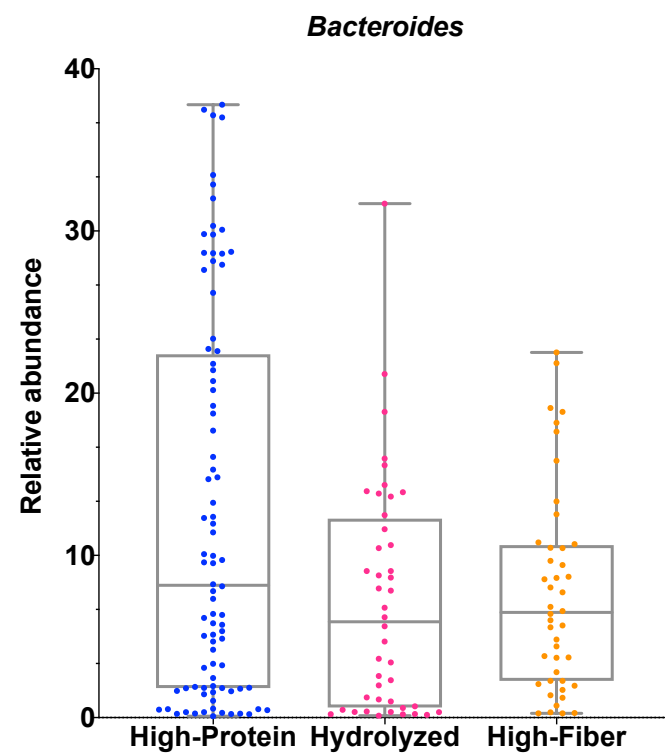**C**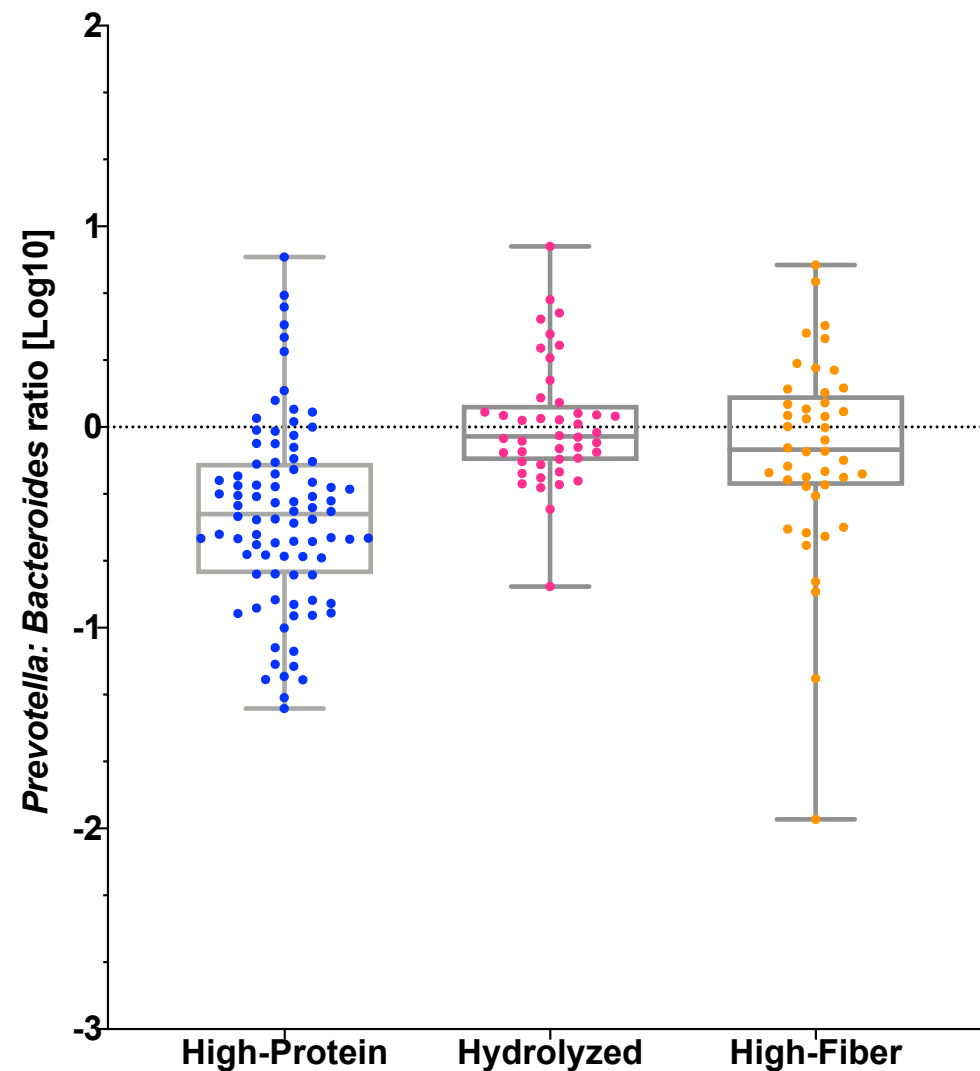

Supplement: Supplementary file 5 — Additional file 5: Figure S4. A: Prevotella and B: Bacteroides relative abundances as a function of the diet. C: Ratios between the two genera in the different categories of diet. [file 42523_2021_101_MOESM5_ESM.pdf]

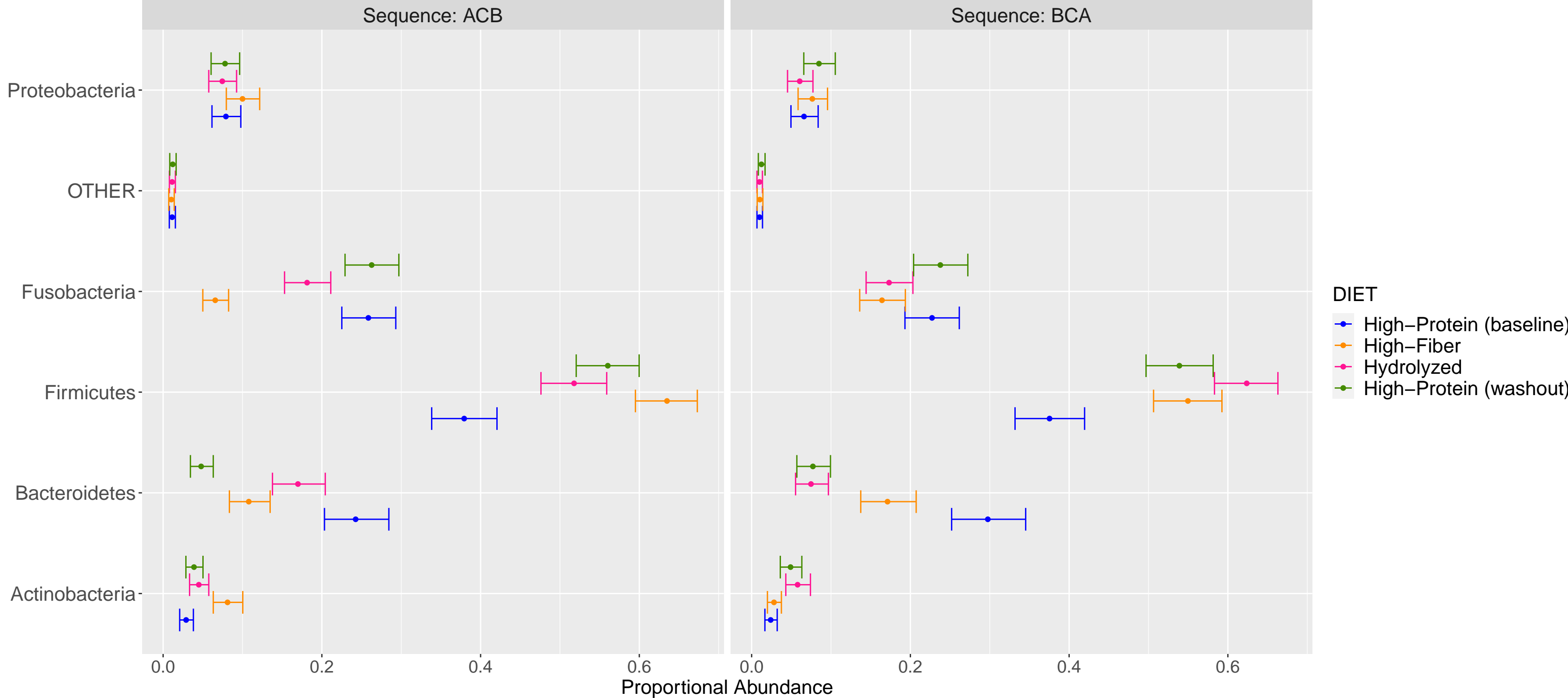

Supplement: Supplementary file 7 — Additional file 7: Figure S5. The posterior estimated population mean of relative abundances at phylum level in diet sequence ACB and BCA. Points are the posterior population mean. The bars are the 89% credible intervals. Inter-subject variation is not included. Baseline [high-protein] (diet C), High-Fiber (diet B), Hydrolyzed (diet A) and Washout [high-protein] (diet C). [file 42523_2021_101_MOESM7_ESM.pdf]

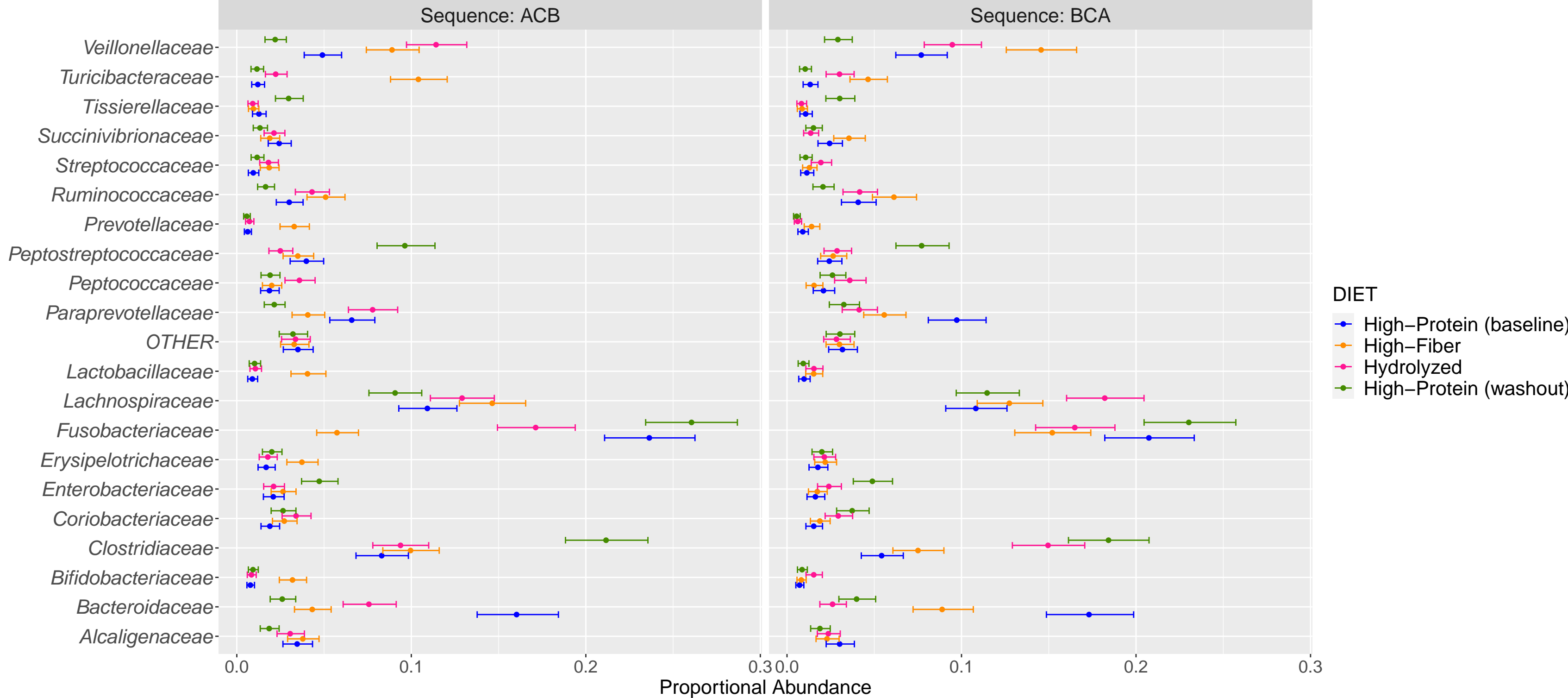

Supplement: Supplementary file 8 — Additional file 8: Figure S6. The posterior estimated population mean of relative abundances at family level in diet sequence ACB and BCA. Top of the 20 most abundant families. Points are the posterior population mean. The bars are the 89% credible intervals. Inter-subject variation is not included. Baseline [high-protein] (diet C), High-Fiber (diet B), Hydrolyzed (diet A) and Washout [high-protein] (diet C). [file 42523_2021_101_MOESM8_ESM.pdf]
