# Supplementary material for: Effect of sequentially fed high protein, hydrolyzed protein, and high fiber diets on the fecal microbiota of healthy dogs: a cross-over study
Source: Anim Microbiome. 2021 Jun 11;3:42. doi: 10.1186/s42523-021-00101-8 (PMC8194187; doi:10.1186/s42523-021-00101-8)
Supplement: Supplementary file 2 — Additional file 2: Table S1. Estimates of the Linear mixed model for Shannon Index. [file 42523_2021_101_MOESM2_ESM.docx]

Table 1: Linear mixed model for Shannon Index.

| PARAMETER | RMLE | LOWER 95% CL | UPPER 95% CL |
| --- | --- | --- | --- |
| Fixed Effects | | | |
| Intercept | 6.60 | 6.39 | 6.81 |
| Diet: High-protein (Baseline) | 0* | - | - |
| Diet: Hydrolyzed | 0.0254 | -0.265 | 0.316 |
| Diet: High-insoluble Fiber | 0.0224 | -0.268 | 0.313 |
| Diet: High-protein (Washout) | -0.755 | -1.05 | -0.464 |
| Sequence (BCA) | -0.108 | -0.411 | 0.194 |
| Diet: Hydrolyzed ^†^ | 0.312 | -0.108 | 0.733 |
| Diet: High-insoluble Fiber^†^ | 0.242 | -0.178 | 0.663 |
| Diet: High-protein^†^  (Washout) | 0.471 | 0.0501 | 0.891 |
| Random Effects | | | |
| Between-subject σ (Intercept) | 0.105 | 0^‡^ | 0.232 |
| Residual σ | 0.512 | 0.444 | 0.564 |

Marginal R^2^: 0.254; Conditional R^2^: 0.285. RMLE: restricted maximum likelihood estimate. CL: confidence limit. *: base level (coefficient set at zero) ^†^: interaction term with sequence BCA. ^‡^: arbitrarily close to zero.
