# Supplementary material for: Effect of sequentially fed high protein, hydrolyzed protein, and high fiber diets on the fecal microbiota of healthy dogs: a cross-over study
Source: Anim Microbiome. 2021 Jun 11;3:42. doi: 10.1186/s42523-021-00101-8 (PMC8194187; doi:10.1186/s42523-021-00101-8)
Supplement: Supplementary file 6 — Additional file 6: Table S2. List of ingredients commercial diets. [file 42523_2021_101_MOESM6_ESM.docx]

Table 2: List of ingredients commercial diets.

| **Diet** | **Ingredients** |
| --- | --- |
| Hill's® Prescription Diet® z/d® Canine**^†^** | Corn Starch, Hydrolyzed Chicken Liver, Soybean Oil, Powdered Cellulose, Calcium  Carbonate, Dicalcium Phosphate, Lactic Acid, Glyceryl Monostearate, Potassium Chloride,  Iodized Salt, Choline Chloride, DL-Methionine, vitamins (Vitamin E Supplement, LAscorbyl-  2-Polyphosphate (source of Vitamin C), Niacin Supplement, Thiamine  Mononitrate, Vitamin A Supplement, Calcium Pantothenate, Biotin, Vitamin B12  Supplement, Pyridoxine Hydrochloride, Riboflavin Supplement, Folic Acid, Vitamin D3  Supplement), minerals (Ferrous Sulfate, Zinc Oxide, Copper sulfate, Manganous Oxide,  Calcium Iodate, Sodium Selenite), Taurine, Mixed Tocopherols for freshness, Natural  Flavors, Beta-Carotene |
| Hill's™ Prescription Diet™ w/d™ Canine^‡^ | Chicken Liver Flavor, Soybean Mill Run, Soybean Oil, Dried Beet Pulp, Lactic Acid,  Soybean Meal, Caramel color, Potassium Chloride, Calcium Sulfate, Flaxseed, L-Lysine,  Choline Chloride, Calcium Carbonate, vitamins (Vitamin E Supplement, L-Ascorbyl-2-  Polyphosphate (source of vitamin C), Niacin Supplement, Thiamine Mononitrate, Vitamin  A Supplement, Calcium Pantothenate, Biotin, Vitamin B12 Supplement, Pyridoxine  Hydrochloride, Riboflavin Supplement, Folic Acid, Vitamin D3 Supplement), Taurine,  minerals (Ferrous Sulfate, Zinc Oxide, Copper Sulfate, Manganous Oxide, Calcium Iodate,  Sodium Selenite), Iodized Salt, L-Tryptophan, L-Carnitine, L-Threonine, Mixed  Tocopherols for freshness, Phosphoric Acid, Beta-Carotene, Natural Flavors. |

**^†^**Diets made prior to August 2016. **^‡^**Diets made prior to April 2015.
