# Supplementary material for: Effect of sequentially fed high protein, hydrolyzed protein, and high fiber diets on the fecal microbiota of healthy dogs: a cross-over study
Source: Anim Microbiome. 2021 Jun 11;3:42. doi: 10.1186/s42523-021-00101-8 (PMC8194187; doi:10.1186/s42523-021-00101-8)
Supplement: Supplementary file 9 — Additional file 9: Table S3. Hierarchical Dirichlet regression model values of the (A) ‘predicted’ and (B) ‘fitted’ interval plots at phylum level. [file 42523_2021_101_MOESM9_ESM.docx]

Table 3A: Hierarchical Dirichlet regression model prediction values of the 'predicted' interval plots.

| **Estimate** | **Est.Error** | **Q5.5** | **Q94.5** | **Family** | **Sequence** | **Diet** |
| --- | --- | --- | --- | --- | --- | --- |
| 0.02877827 | 0.03775055 | 1.80E-04 | 0.10204479 | Actinobacteria | ACB | Baseline (High-Protein) |
| 0.04470482 | 0.04642554 | 1.61E-03 | 0.13434001 | Actinobacteria | ACB | Hydrolyzed |
| 0.08062569 | 0.06154416 | 1.04E-02 | 0.19571735 | Actinobacteria | ACB | High-insoluble Fiber |
| 0.03851988 | 0.04347245 | 8.59E-04 | 0.12328041 | Actinobacteria | ACB | Washout (High-Protein) |
| 0.02402137 | 0.03454163 | 5.08E-05 | 0.08944909 | Actinobacteria | BCA | Baseline (High-Protein) |
| 0.05824634 | 0.0531612 | 3.89E-03 | 0.16037523 | Actinobacteria | BCA | Hydrolyzed |
| 0.02841344 | 0.037527 | 1.48E-04 | 0.10161796 | Actinobacteria | BCA | High-insoluble Fiber |
| 0.0491605 | 0.04862046 | 2.08E-03 | 0.14352859 | Actinobacteria | BCA | Washout (High-Protein) |
| 0.24202427 | 0.09770514 | 1.01E-01 | 0.4121071 | Bacteroidetes | ACB | Baseline (High-Protein) |
| 0.16977614 | 0.08601988 | 5.30E-02 | 0.32210491 | Bacteroidetes | ACB | Hydrolyzed |
| 0.10821696 | 0.07084532 | 2.15E-02 | 0.23961858 | Bacteroidetes | ACB | High-insoluble Fiber |
| 0.04798578 | 0.04869524 | 1.91E-03 | 0.14312185 | Bacteroidetes | ACB | Washout (High-Protein) |
| 0.29665857 | 0.10541169 | 1.39E-01 | 0.47807247 | Bacteroidetes | BCA | Baseline (High-Protein) |
| 0.0755757 | 0.05999314 | 8.17E-03 | 0.18886234 | Bacteroidetes | BCA | Hydrolyzed |
| 0.17046896 | 0.08654575 | 5.32E-02 | 0.32574498 | Bacteroidetes | BCA | High-insoluble Fiber |
| 0.0769349 | 0.06091209 | 9.18E-03 | 0.19200708 | Bacteroidetes | BCA | Washout (High-Protein) |
| 0.38063956 | 0.11037164 | 2.09E-01 | 0.56447034 | Firmicutes | ACB | Baseline (High-Protein) |
| 0.51710264 | 0.11319386 | 3.35E-01 | 0.69736375 | Firmicutes | ACB | Hydrolyzed |
| 0.63562137 | 0.10952025 | 4.53E-01 | 0.80325595 | Firmicutes | ACB | High-insoluble Fiber |
| 0.55988859 | 0.11288475 | 3.75E-01 | 0.73807605 | Firmicutes | ACB | Washout (High-Protein) |
| 0.37616715 | 0.10984507 | 2.06E-01 | 0.55770902 | Firmicutes | BCA | Baseline (High-Protein) |
| 0.62317392 | 0.11066816 | 4.38E-01 | 0.79526278 | Firmicutes | BCA | Hydrolyzed |
| 0.54952256 | 0.11371487 | 3.64E-01 | 0.72939358 | Firmicutes | BCA | High-insoluble Fiber |
| 0.53950027 | 0.11402218 | 3.55E-01 | 0.72090274 | Firmicutes | BCA | Washout (High-Protein) |
| 0.258424 | 0.09912801 | 1.14E-01 | 0.43003089 | Fusobacteria | ACB | Baseline (High-Protein) |
| 0.1829914 | 0.08771269 | 6.20E-02 | 0.33986898 | Fusobacteria | ACB | Hydrolyzed |
| 0.065493 | 0.05618068 | 5.72E-03 | 0.17310491 | Fusobacteria | ACB | High-insoluble Fiber |
| 0.26245598 | 0.10018578 | 1.17E-01 | 0.43606959 | Fusobacteria | ACB | Washout (High-Protein) |
| 0.22710087 | 0.09512025 | 9.18E-02 | 0.39322691 | Fusobacteria | BCA | Baseline (High-Protein) |
| 0.17327103 | 0.08596384 | 5.60E-02 | 0.32652269 | Fusobacteria | BCA | Hydrolyzed |
| 0.16429582 | 0.0839809 | 5.02E-02 | 0.31452554 | Fusobacteria | BCA | High-insoluble Fiber |
| 0.23719091 | 0.09662762 | 9.82E-02 | 0.40648036 | Fusobacteria | BCA | Washout (High-Protein) |
| 0.07869252 | 0.06136205 | 9.43E-03 | 0.19444399 | Proteobacteria | ACB | Baseline (High-Protein) |
| 0.07400181 | 0.05895457 | 8.50E-03 | 0.18774343 | Proteobacteria | ACB | Hydrolyzed |
| 0.09967377 | 0.06829047 | 1.80E-02 | 0.22744997 | Proteobacteria | ACB | High-insoluble Fiber |
| 0.07890352 | 0.06109178 | 9.76E-03 | 0.194189 | Proteobacteria | ACB | Washout (High-Protein) |
| 0.06590506 | 0.05603049 | 5.93E-03 | 0.17266357 | Proteobacteria | BCA | Baseline (High-Protein) |
| 0.0596186 | 0.05327044 | 4.42E-03 | 0.16111454 | Proteobacteria | BCA | Hydrolyzed |
| 0.07689317 | 0.06030579 | 8.85E-03 | 0.19160537 | Proteobacteria | BCA | High-insoluble Fiber |
| 0.08519309 | 0.06364608 | 1.16E-02 | 0.20641853 | Proteobacteria | BCA | Washout (High-Protein) |
| 0.01144139 | 0.02379409 | 2.42E-08 | 0.05455958 | OTHER | ACB | Baseline (High-Protein) |
| 0.01142319 | 0.02351513 | 3.43E-08 | 0.0536143 | OTHER | ACB | Hydrolyzed |
| 0.01036921 | 0.02297114 | 5.79E-09 | 0.05067257 | OTHER | ACB | High-insoluble Fiber |
| 0.01224626 | 0.02473805 | 7.41E-08 | 0.0570854 | OTHER | ACB | Washout (High-Protein) |
| 0.01014697 | 0.02210182 | 2.75E-09 | 0.05013547 | OTHER | BCA | Baseline (High-Protein) |
| 0.0101144 | 0.02300433 | 2.05E-09 | 0.04950258 | OTHER | BCA | Hydrolyzed |
| 0.01040606 | 0.02253889 | 6.72E-09 | 0.05113902 | OTHER | BCA | High-insoluble Fiber |
| 0.01202032 | 0.02386258 | 1.03E-07 | 0.05646556 | OTHER | BCA | Washout (High-Protein) |

Table 3B: Hierarchical Dirichlet regression model prediction values of the 'fitted' interval plots.

| **Estimate** | **Est.Error** | **Q5.5** | **Q94.5** | **Family** | **Sequence** | **Diet** |
| --- | --- | --- | --- | --- | --- | --- |
| 0.029004528 | 0.005419066 | 0.020869928 | 0.03815259 | Actinobacteria | ACB | Baseline (High-Protein) |
| 0.04496957 | 0.007633667 | 0.033327825 | 0.05753268 | Actinobacteria | ACB | Hydrolyzed |
| 0.081171523 | 0.011681153 | 0.063214607 | 0.10036967 | Actinobacteria | ACB | High-insoluble Fiber |
| 0.038900574 | 0.006818066 | 0.028705778 | 0.05023387 | Actinobacteria | ACB | Washout (High-Protein) |
| 0.02395454 | 0.004874103 | 0.016685945 | 0.03220956 | Actinobacteria | BCA | Baseline (High-Protein) |
| 0.057892449 | 0.009660054 | 0.043028249 | 0.07408983 | Actinobacteria | BCA | Hydrolyzed |
| 0.028157385 | 0.005544801 | 0.019884959 | 0.03748894 | Actinobacteria | BCA | High-insoluble Fiber |
| 0.049009188 | 0.008565516 | 0.036083344 | 0.06323087 | Actinobacteria | BCA | Washout (High-Protein) |
| 0.242570904 | 0.025232354 | 0.203303779 | 0.28442127 | Bacteroidetes | ACB | Baseline (High-Protein) |
| 0.169878673 | 0.020869393 | 0.137799837 | 0.2043142 | Bacteroidetes | ACB | Hydrolyzed |
| 0.107919202 | 0.016075066 | 0.083513784 | 0.13481891 | Bacteroidetes | ACB | High-insoluble Fiber |
| 0.047916066 | 0.009008205 | 0.034392536 | 0.06318809 | Bacteroidetes | ACB | Washout (High-Protein) |
| 0.297560923 | 0.029155512 | 0.251991687 | 0.34528331 | Bacteroidetes | BCA | Baseline (High-Protein) |
| 0.07476901 | 0.012934084 | 0.055362103 | 0.09667812 | Bacteroidetes | BCA | Hydrolyzed |
| 0.171200635 | 0.021829591 | 0.137447164 | 0.20729736 | Bacteroidetes | BCA | High-insoluble Fiber |
| 0.077171799 | 0.013277714 | 0.05693916 | 0.0993336 | Bacteroidetes | BCA | Washout (High-Protein) |
| 0.379316109 | 0.025634611 | 0.338406021 | 0.42066395 | Firmicutes | ACB | Baseline (High-Protein) |
| 0.517780911 | 0.025910407 | 0.476067843 | 0.55893179 | Firmicutes | ACB | Hydrolyzed |
| 0.634886776 | 0.024370575 | 0.595091589 | 0.67304967 | Firmicutes | ACB | High-insoluble Fiber |
| 0.560297267 | 0.024802947 | 0.520492705 | 0.59977618 | Firmicutes | ACB | Washout (High-Protein) |
| 0.375335065 | 0.027459765 | 0.331871231 | 0.41950646 | Firmicutes | BCA | Baseline (High-Protein) |
| 0.623765607 | 0.02493218 | 0.583135692 | 0.66304991 | Firmicutes | BCA | Hydrolyzed |
| 0.54968594 | 0.0269323 | 0.506408078 | 0.59254258 | Firmicutes | BCA | High-insoluble Fiber |
| 0.538927644 | 0.026466324 | 0.496914397 | 0.58149844 | Firmicutes | BCA | Washout (High-Protein) |
| 0.258615033 | 0.021297735 | 0.225153288 | 0.29314931 | Fusobacteria | ACB | Baseline (High-Protein) |
| 0.181408414 | 0.018282922 | 0.153008606 | 0.21122052 | Fusobacteria | ACB | Hydrolyzed |
| 0.065670611 | 0.010062409 | 0.050023711 | 0.08250881 | Fusobacteria | ACB | High-insoluble Fiber |
| 0.262755662 | 0.021246539 | 0.229210066 | 0.29702506 | Fusobacteria | ACB | Washout (High-Protein) |
| 0.227278773 | 0.021411925 | 0.193170946 | 0.26160332 | Fusobacteria | BCA | Baseline (High-Protein) |
| 0.173124291 | 0.018375523 | 0.144211801 | 0.20318472 | Fusobacteria | BCA | Hydrolyzed |
| 0.164221319 | 0.01803025 | 0.136152745 | 0.19366924 | Fusobacteria | BCA | High-insoluble Fiber |
| 0.237749874 | 0.021457853 | 0.204063281 | 0.2723851 | Fusobacteria | BCA | Washout (High-Protein) |
| 0.07912662 | 0.011467795 | 0.06156318 | 0.09787557 | Proteobacteria | ACB | Baseline (High-Protein) |
| 0.074550673 | 0.010959186 | 0.05751664 | 0.09256143 | Proteobacteria | ACB | Hydrolyzed |
| 0.10003413 | 0.013162329 | 0.079644655 | 0.12167126 | Proteobacteria | ACB | High-insoluble Fiber |
| 0.078018362 | 0.011265626 | 0.060444284 | 0.09644236 | Proteobacteria | ACB | Washout (High-Protein) |
| 0.065950982 | 0.010714054 | 0.049529171 | 0.08376709 | Proteobacteria | BCA | Baseline (High-Protein) |
| 0.060570947 | 0.010017238 | 0.045195119 | 0.07723788 | Proteobacteria | BCA | Hydrolyzed |
| 0.076433121 | 0.011602881 | 0.058515793 | 0.09560655 | Proteobacteria | BCA | High-insoluble Fiber |
| 0.084810318 | 0.012418138 | 0.065688687 | 0.10536895 | Proteobacteria | BCA | Washout (High-Protein) |
| 0.011366806 | 0.002394278 | 0.007821228 | 0.01545626 | OTHER | ACB | Baseline (High-Protein) |
| 0.011411759 | 0.002368269 | 0.007923998 | 0.0154419 | OTHER | ACB | Hydrolyzed |
| 0.010317759 | 0.002172687 | 0.007112347 | 0.01400374 | OTHER | ACB | High-insoluble Fiber |
| 0.012112068 | 0.002532438 | 0.008377943 | 0.01639771 | OTHER | ACB | Washout (High-Protein) |
| 0.009919717 | 0.00218787 | 0.006696185 | 0.01366758 | OTHER | BCA | Baseline (High-Protein) |
| 0.009877697 | 0.002177632 | 0.006681328 | 0.01357856 | OTHER | BCA | Hydrolyzed |
| 0.0103016 | 0.002275248 | 0.006976944 | 0.0141665 | OTHER | BCA | High-insoluble Fiber |
| 0.012331177 | 0.002661881 | 0.008416957 | 0.0169016 | OTHER | BCA | Washout (High-Protein) |
