# Supplementary material for: Effect of sequentially fed high protein, hydrolyzed protein, and high fiber diets on the fecal microbiota of healthy dogs: a cross-over study
Source: Anim Microbiome. 2021 Jun 11;3:42. doi: 10.1186/s42523-021-00101-8 (PMC8194187; doi:10.1186/s42523-021-00101-8)
Supplement: Supplementary file 10 — Additional file 10: Table S4. Hierarchical Dirichlet regression model values of the (A) ‘predicted’ and (B) ‘fitted’ interval plots at family level. [file 42523_2021_101_MOESM10_ESM.docx]

Table 4A: Hierarchical Dirichlet regression model prediction values of the 'predicted' interval plots.

| **Estimate** | **Est.Error** | **Q5.5** | **Q94.5** | **Family** | **Sequence** | **Diet** |
| --- | --- | --- | --- | --- | --- | --- |
| 0.00772835 | 0.01453422 | 3.49E-07 | 0.03455497 | *Bifidobacteriaceae* | ACB | Baseline (High-Protein) |
| 0.00835039 | 0.01510549 | 7.77E-07 | 0.03613132 | *Bifidobacteriaceae* | ACB | Hydrolyzed |
| 0.03199095 | 0.0296294 | 2.26E-03 | 0.0888233 | *Bifidobacteriaceae* | ACB | High-insoluble Fiber |
| 0.00934189 | 0.01623334 | 1.84E-06 | 0.03952158 | *Bifidobacteriaceae* | ACB | Washout (High-Protein) |
| 0.00719315 | 0.01411085 | 1.30E-07 | 0.03283147 | *Bifidobacteriaceae* | BCA | Baseline (High-Protein) |
| 0.01549457 | 0.02074314 | 8.30E-05 | 0.05533793 | *Bifidobacteriaceae* | BCA | Hydrolyzed |
| 0.00818332 | 0.01495408 | 6.10E-07 | 0.03563648 | *Bifidobacteriaceae* | BCA | High-insoluble Fiber |
| 0.00879617 | 0.01544713 | 1.05E-06 | 0.03786627 | *Bifidobacteriaceae* | BCA | Washout (High-Protein) |
| 0.01896102 | 0.02277215 | 2.70E-04 | 0.06311912 | *Coriobacteriaceae* | ACB | Baseline (High-Protein) |
| 0.03424073 | 0.03066034 | 2.59E-03 | 0.0913491 | *Coriobacteriaceae* | ACB | Hydrolyzed |
| 0.02740324 | 0.02751611 | 1.31E-03 | 0.08107727 | *Coriobacteriaceae* | ACB | High-insoluble Fiber |
| 0.02631551 | 0.02689752 | 1.12E-03 | 0.07816416 | *Coriobacteriaceae* | ACB | Washout (High-Protein) |
| 0.01536128 | 0.02065676 | 7.73E-05 | 0.05566576 | *Coriobacteriaceae* | BCA | Baseline (High-Protein) |
| 0.02927294 | 0.02842938 | 1.72E-03 | 0.08437022 | *Coriobacteriaceae* | BCA | Hydrolyzed |
| 0.01899522 | 0.02278129 | 2.53E-04 | 0.06363343 | *Coriobacteriaceae* | BCA | High-insoluble Fiber |
| 0.03721603 | 0.03168255 | 3.53E-03 | 0.09727029 | *Coriobacteriaceae* | BCA | Washout (High-Protein) |
| 0.16065794 | 0.06275942 | 7.19E-02 | 0.2700728 | *Bacteroidaceae* | ACB | Baseline (High-Protein) |
| 0.07577241 | 0.04483343 | 1.93E-02 | 0.15863598 | *Bacteroidaceae* | ACB | Hydrolyzed |
| 0.04307019 | 0.03432213 | 5.13E-03 | 0.10819335 | *Bacteroidaceae* | ACB | High-insoluble Fiber |
| 0.02602406 | 0.02681119 | 1.10E-03 | 0.07716552 | *Bacteroidaceae* | ACB | Washout (High-Protein) |
| 0.17263019 | 0.06429038 | 8.02E-02 | 0.28480279 | *Bacteroidaceae* | BCA | Baseline (High-Protein) |
| 0.02593396 | 0.02659778 | 1.03E-03 | 0.07721622 | *Bacteroidaceae* | BCA | Hydrolyzed |
| 0.08895866 | 0.04836627 | 2.65E-02 | 0.1767508 | *Bacteroidaceae* | BCA | High-insoluble Fiber |
| 0.04000533 | 0.03291362 | 4.31E-03 | 0.10251875 | *Bacteroidaceae* | BCA | Washout (High-Protein) |
| 0.00630952 | 0.0132089 | 1.76E-08 | 0.02979099 | *Prevotellaceae* | ACB | Baseline (High-Protein) |
| 0.00731511 | 0.0145367 | 1.22E-07 | 0.03312346 | *Prevotellaceae* | ACB | Hydrolyzed |
| 0.03263725 | 0.02970772 | 2.35E-03 | 0.08914332 | *Prevotellaceae* | ACB | High-insoluble Fiber |
| 0.00562496 | 0.01230212 | 3.15E-09 | 0.02759574 | *Prevotellaceae* | ACB | Washout (High-Protein) |
| 0.00907943 | 0.01577807 | 1.14E-06 | 0.03846674 | *Prevotellaceae* | BCA | Baseline (High-Protein) |
| 0.00617157 | 0.01291185 | 1.29E-08 | 0.02979924 | *Prevotellaceae* | BCA | Hydrolyzed |
| 0.01387876 | 0.01970406 | 4.23E-05 | 0.0515494 | *Prevotellaceae* | BCA | High-insoluble Fiber |
| 0.00568337 | 0.01259284 | 2.71E-09 | 0.02855995 | *Prevotellaceae* | BCA | Washout (High-Protein) |
| 0.06579631 | 0.04188499 | 1.45E-02 | 0.14402231 | *Paraprevotellaceae* | ACB | Baseline (High-Protein) |
| 0.07780424 | 0.04510103 | 2.08E-02 | 0.16108719 | *Paraprevotellaceae* | ACB | Hydrolyzed |
| 0.04069888 | 0.03311171 | 4.58E-03 | 0.10270585 | *Paraprevotellaceae* | ACB | High-insoluble Fiber |
| 0.02132575 | 0.02414667 | 4.88E-04 | 0.0678168 | *Paraprevotellaceae* | ACB | Washout (High-Protein) |
| 0.09709371 | 0.049426 | 3.12E-02 | 0.18629352 | *Paraprevotellaceae* | BCA | Baseline (High-Protein) |
| 0.04134157 | 0.03337293 | 4.59E-03 | 0.10481629 | *Paraprevotellaceae* | BCA | Hydrolyzed |
| 0.05562141 | 0.038372 | 1.02E-02 | 0.12708537 | *Paraprevotellaceae* | BCA | High-insoluble Fiber |
| 0.03257985 | 0.02968866 | 2.37E-03 | 0.08934557 | *Paraprevotellaceae* | BCA | Washout (High-Protein) |
| 0.00892465 | 0.01560658 | 1.35E-06 | 0.03780231 | *Lactobacillaceae* | ACB | Baseline (High-Protein) |
| 0.01068025 | 0.01699914 | 5.75E-06 | 0.04357655 | *Lactobacillaceae* | ACB | Hydrolyzed |
| 0.0406488 | 0.03334088 | 4.48E-03 | 0.10428722 | *Lactobacillaceae* | ACB | High-insoluble Fiber |
| 0.01033787 | 0.01707066 | 3.79E-06 | 0.04162096 | *Lactobacillaceae* | ACB | Washout (High-Protein) |
| 0.00982595 | 0.01636907 | 2.95E-06 | 0.0409108 | *Lactobacillaceae* | BCA | Baseline (High-Protein) |
| 0.01536288 | 0.02036671 | 8.22E-05 | 0.05464366 | *Lactobacillaceae* | BCA | Hydrolyzed |
| 0.01530353 | 0.02021379 | 7.57E-05 | 0.0543929 | *Lactobacillaceae* | BCA | High-insoluble Fiber |
| 0.00933916 | 0.01611188 | 1.79E-06 | 0.03968763 | *Lactobacillaceae* | BCA | Washout (High-Protein) |
| 0.00945851 | 0.01626035 | 2.37E-06 | 0.03980861 | *Streptococcaceae* | ACB | Baseline (High-Protein) |
| 0.01846352 | 0.02255832 | 2.01E-04 | 0.06268032 | *Streptococcaceae* | ACB | Hydrolyzed |
| 0.01852219 | 0.02254455 | 2.43E-04 | 0.06208654 | *Streptococcaceae* | ACB | High-insoluble Fiber |
| 0.01169632 | 0.01764495 | 1.26E-05 | 0.04530172 | *Streptococcaceae* | ACB | Washout (High-Protein) |
| 0.01105771 | 0.01716523 | 1.04E-05 | 0.04385788 | *Streptococcaceae* | BCA | Baseline (High-Protein) |
| 0.01962711 | 0.02320674 | 2.75E-04 | 0.06440584 | *Streptococcaceae* | BCA | Hydrolyzed |
| 0.01292719 | 0.01904323 | 2.57E-05 | 0.0488732 | *Streptococcaceae* | BCA | High-insoluble Fiber |
| 0.01077595 | 0.017209 | 6.25E-06 | 0.04399052 | *Streptococcaceae* | BCA | Washout (High-Protein) |
| 0.01207399 | 0.01819571 | 1.57E-05 | 0.0469059 | *Turicibacteraceae* | ACB | Baseline (High-Protein) |
| 0.02211506 | 0.02427397 | 5.54E-04 | 0.06876037 | *Turicibacteraceae* | ACB | Hydrolyzed |
| 0.10341292 | 0.05100205 | 3.53E-02 | 0.19502943 | *Turicibacteraceae* | ACB | High-insoluble Fiber |
| 0.01155394 | 0.01796046 | 1.33E-05 | 0.04556866 | *Turicibacteraceae* | ACB | Washout (High-Protein) |
| 0.01310019 | 0.01895862 | 3.20E-05 | 0.0492082 | *Turicibacteraceae* | BCA | Baseline (High-Protein) |
| 0.03034562 | 0.02894101 | 1.75E-03 | 0.08532811 | *Turicibacteraceae* | BCA | Hydrolyzed |
| 0.04648191 | 0.03540238 | 6.39E-03 | 0.11239242 | *Turicibacteraceae* | BCA | High-insoluble Fiber |
| 0.01044881 | 0.01688125 | 5.29E-06 | 0.04299117 | *Turicibacteraceae* | BCA | Washout (High-Protein) |
| 0.08357515 | 0.04642534 | 2.33E-02 | 0.16813664 | *Clostridiaceae* | ACB | Baseline (High-Protein) |
| 0.09357945 | 0.04901174 | 2.92E-02 | 0.18151022 | *Clostridiaceae* | ACB | Hydrolyzed |
| 0.09959069 | 0.05055193 | 3.23E-02 | 0.1907985 | *Clostridiaceae* | ACB | High-insoluble Fiber |
| 0.21196572 | 0.06994241 | 1.10E-01 | 0.33193794 | *Clostridiaceae* | ACB | Washout (High-Protein) |
| 0.05467557 | 0.03823165 | 9.37E-03 | 0.12478776 | *Clostridiaceae* | BCA | Baseline (High-Protein) |
| 0.1496231 | 0.06030665 | 6.42E-02 | 0.25525683 | *Clostridiaceae* | BCA | Hydrolyzed |
| 0.0753823 | 0.04436351 | 1.93E-02 | 0.1567356 | *Clostridiaceae* | BCA | High-insoluble Fiber |
| 0.18369945 | 0.06505067 | 8.92E-02 | 0.29599448 | *Clostridiaceae* | BCA | Washout (High-Protein) |
| 0.10903077 | 0.05237319 | 3.85E-02 | 0.20301908 | *Lachnospiraceae* | ACB | Baseline (High-Protein) |
| 0.12919348 | 0.05636283 | 5.21E-02 | 0.22809554 | *Lachnospiraceae* | ACB | Hydrolyzed |
| 0.146155 | 0.05938026 | 6.34E-02 | 0.2512779 | *Lachnospiraceae* | ACB | High-insoluble Fiber |
| 0.09104228 | 0.04833991 | 2.72E-02 | 0.17744192 | *Lachnospiraceae* | ACB | Washout (High-Protein) |
| 0.10797913 | 0.05268075 | 3.76E-02 | 0.2018357 | *Lachnospiraceae* | BCA | Baseline (High-Protein) |
| 0.1812958 | 0.06503917 | 8.67E-02 | 0.29285349 | *Lachnospiraceae* | BCA | Hydrolyzed |
| 0.12771935 | 0.05620786 | 4.99E-02 | 0.22633829 | *Lachnospiraceae* | BCA | High-insoluble Fiber |
| 0.11508178 | 0.05379046 | 4.23E-02 | 0.21131715 | *Lachnospiraceae* | BCA | Washout (High-Protein) |
| 0.01831468 | 0.02232483 | 2.57E-04 | 0.06092799 | *Peptococcaceae* | ACB | Baseline (High-Protein) |
| 0.03567091 | 0.03116992 | 3.03E-03 | 0.09495634 | *Peptococcaceae* | ACB | Hydrolyzed |
| 0.02023697 | 0.02347083 | 3.76E-04 | 0.06569193 | *Peptococcaceae* | ACB | High-insoluble Fiber |
| 0.01867661 | 0.02217867 | 2.83E-04 | 0.06212349 | *Peptococcaceae* | ACB | Washout (High-Potein) |
| 0.02096589 | 0.02395639 | 3.83E-04 | 0.0669425 | *Peptococcaceae* | BCA | Baseline (High-Potein) |
| 0.03614864 | 0.03142607 | 3.16E-03 | 0.09604025 | *Peptococcaceae* | BCA | Hydrolyzed |
| 0.01569686 | 0.02084851 | 9.01E-05 | 0.05511641 | *Peptococcaceae* | BCA | High-insoluble Fiber |
| 0.02626085 | 0.02691506 | 9.87E-04 | 0.07859204 | *Peptococcaceae* | BCA | Washout (High-Potein) |
| 0.03961496 | 0.03295066 | 4.23E-03 | 0.10270468 | *Peptostreptococcaceae* | ACB | Baseline (High-Potein) |
| 0.02466989 | 0.02563298 | 9.01E-04 | 0.07406259 | *Peptostreptococcaceae* | ACB | Hydrolyzed |
| 0.0346147 | 0.03070622 | 2.84E-03 | 0.09228565 | *Peptostreptococcaceae* | ACB | High-insoluble Fiber |
| 0.09600453 | 0.05001422 | 2.99E-02 | 0.18651307 | *Peptostreptococcaceae* | ACB | Washout (High-Potein) |
| 0.0242382 | 0.02566104 | 7.65E-04 | 0.07346749 | *Peptostreptococcaceae* | BCA | Baseline (High-Potein) |
| 0.02863485 | 0.02824167 | 1.46E-03 | 0.08348317 | *Peptostreptococcaceae* | BCA | Hydrolyzed |
| 0.02645439 | 0.02682507 | 1.12E-03 | 0.0776294 | *Peptostreptococcaceae* | BCA | High-insoluble Fiber |
| 0.07678119 | 0.04507142 | 2.00E-02 | 0.15978881 | *Peptostreptococcaceae* | BCA | Washout (High-Protein) |
| 0.02986096 | 0.02862228 | 1.80E-03 | 0.08312897 | *Ruminococcaceae* | ACB | Baseline (High-Protein) |
| 0.04324502 | 0.03426557 | 5.49E-03 | 0.10868913 | *Ruminococcaceae* | ACB | Hydrolyzed |
| 0.05056664 | 0.03681812 | 8.36E-03 | 0.11957364 | *Ruminococcaceae* | ACB | High-insoluble Fiber |
| 0.01636096 | 0.02117251 | 1.32E-04 | 0.05749093 | *Ruminococcaceae* | ACB | Washout (High-Protein) |
| 0.04138 | 0.03406726 | 4.60E-03 | 0.10503764 | *Ruminococcaceae* | BCA | Baseline (High-Protein) |
| 0.04210692 | 0.03411687 | 4.90E-03 | 0.10649482 | *Ruminococcaceae* | BCA | Hydrolyzed |
| 0.0610015 | 0.04020408 | 1.22E-02 | 0.13495162 | *Ruminococcaceae* | BCA | High-insoluble Fiber |
| 0.02059744 | 0.02327848 | 4.06E-04 | 0.06642499 | *Ruminococcaceae* | BCA | Washout (High-Protein) |
| 0.04924876 | 0.03647646 | 7.57E-03 | 0.11670674 | *Veillonellaceae* | ACB | Baseline (High-Protein) |
| 0.11429254 | 0.05387784 | 4.14E-02 | 0.21103823 | *Veillonellaceae* | ACB | Hydrolyzed |
| 0.08920934 | 0.04778497 | 2.66E-02 | 0.17563918 | *Veillonellaceae* | ACB | High-insoluble Fiber |
| 0.02169378 | 0.02416314 | 5.30E-04 | 0.06873595 | *Veillonellaceae* | ACB | Washout (High-Protein) |
| 0.07730785 | 0.04561989 | 1.99E-02 | 0.16182266 | *Veillonellaceae* | BCA | Baseline (High-Protein) |
| 0.09453537 | 0.04896668 | 2.93E-02 | 0.18229535 | *Veillonellaceae* | BCA | Hydrolyzed |
| 0.14538222 | 0.05947755 | 6.19E-02 | 0.25069438 | *Veillonellaceae* | BCA | High-insoluble Fiber |
| 0.02954052 | 0.02849754 | 1.61E-03 | 0.08370769 | *Veillonellaceae* | BCA | Washout (High-Protein) |
| 0.01244655 | 0.01830787 | 2.31E-05 | 0.04800938 | *Tissierellaceae* | ACB | Baseline (High-Protein) |
| 0.00911332 | 0.01586824 | 1.50E-06 | 0.03883452 | *Tissierellaceae* | ACB | Hydrolyzed |
| 0.00950992 | 0.0159753 | 2.27E-06 | 0.04017545 | *Tissierellaceae* | ACB | High-insoluble Fiber |
| 0.02984292 | 0.02854936 | 1.67E-03 | 0.08506534 | *Tissierellaceae* | ACB | Washout (High-Protein) |
| 0.01059017 | 0.01703656 | 5.88E-06 | 0.04360482 | *Tissierellaceae* | BCA | Baseline (High-Protein) |
| 0.00829395 | 0.01493814 | 5.08E-07 | 0.03613201 | *Tissierellaceae* | BCA | Hydrolyzed |
| 0.00854972 | 0.01506595 | 8.96E-07 | 0.03662149 | *Tissierellaceae* | BCA | High-insoluble Fiber |
| 0.0301698 | 0.0286788 | 1.85E-03 | 0.08437019 | *Tissierellaceae* | BCA | Washout (High-Protein) |
| 0.01676274 | 0.02145859 | 1.46E-04 | 0.05752532 | *Erysipelotrichaceae* | ACB | Baseline (High-Protein) |
| 0.01790733 | 0.02244372 | 1.86E-04 | 0.0602777 | *Erysipelotrichaceae* | ACB | Hydrolyzed |
| 0.03726314 | 0.03174806 | 3.60E-03 | 0.09669946 | *Erysipelotrichaceae* | ACB | High-insoluble Fiber |
| 0.02006321 | 0.0233574 | 3.64E-04 | 0.06531674 | *Erysipelotrichaceae* | ACB | Washout (High-Protein) |
| 0.01760089 | 0.02215789 | 1.62E-04 | 0.0603805 | *Erysipelotrichaceae* | BCA | Baseline (High-Protein) |
| 0.02128033 | 0.02413091 | 4.71E-04 | 0.0683119 | *Erysipelotrichaceae* | BCA | Hydrolyzed |
| 0.02172687 | 0.02438824 | 5.19E-04 | 0.06867521 | *Erysipelotrichaceae* | BCA | High-insoluble Fiber |
| 0.02008719 | 0.02358511 | 3.34E-04 | 0.06508438 | *Erysipelotrichaceae* | BCA | Washout (High-Protein) |
| 0.23662016 | 0.07153724 | 1.31E-01 | 0.3594185 | *Fusobacteriaceae* | ACB | Baseline (High-Protein) |
| 0.17088302 | 0.06399495 | 7.95E-02 | 0.28254303 | *Fusobacteriaceae* | ACB | Hydrolyzed |
| 0.05759255 | 0.03891339 | 1.06E-02 | 0.12960613 | *Fusobacteriaceae* | ACB | High-insoluble Fiber |
| 0.26089833 | 0.07419577 | 1.49E-01 | 0.38717572 | *Fusobacteriaceae* | ACB | Washout (High-Protein) |
| 0.20739291 | 0.06902707 | 1.05E-01 | 0.32622536 | *Fusobacteriaceae* | BCA | Baseline (High-Protein) |
| 0.1653672 | 0.06269954 | 7.64E-02 | 0.2744117 | *Fusobacteriaceae* | BCA | Hydrolyzed |
| 0.15164199 | 0.06048518 | 6.60E-02 | 0.25677018 | *Fusobacteriaceae* | BCA | High-insoluble Fiber |
| 0.23002157 | 0.07123187 | 1.25E-01 | 0.35122715 | *Fusobacteriaceae* | BCA | Washout (High-Protein) |
| 0.03456257 | 0.030617 | 2.87E-03 | 0.09312637 | *Alcaligenaceae* | ACB | Baseline (High-Protein) |
| 0.03099576 | 0.02899409 | 1.94E-03 | 0.08700836 | *Alcaligenaceae* | ACB | Hydrolyzed |
| 0.037984 | 0.0322042 | 3.62E-03 | 0.0997755 | *Alcaligenaceae* | ACB | High-insoluble Fiber |
| 0.01869692 | 0.02298813 | 2.25E-04 | 0.06316272 | *Alcaligenaceae* | ACB | Washout (High-Protein) |
| 0.03033963 | 0.02884776 | 1.85E-03 | 0.08546186 | *Alcaligenaceae* | BCA | Baseline (High-Protein) |
| 0.0233979 | 0.02524023 | 7.43E-04 | 0.07235187 | *Alcaligenaceae* | BCA | Hydrolyzed |
| 0.02282547 | 0.02483255 | 6.12E-04 | 0.07079189 | *Alcaligenaceae* | BCA | High-insoluble Fiber |
| 0.01890399 | 0.02295146 | 2.52E-04 | 0.06298715 | *Alcaligenaceae* | BCA | Washout (High-Protein) |
| 0.02420609 | 0.02579585 | 7.83E-04 | 0.07388582 | *Succinivibrionaceae* | ACB | Baseline (High-Protein) |
| 0.02087901 | 0.02356567 | 5.07E-04 | 0.06545146 | *Succinivibrionaceae* | ACB | Hydrolyzed |
| 0.01873991 | 0.02262909 | 2.71E-04 | 0.0628116 | *Succinivibrionaceae* | ACB | High-insoluble Fiber |
| 0.01336612 | 0.01948458 | 3.02E-05 | 0.05060533 | *Succinivibrionaceae* | ACB | Washout (High-Potein) |
| 0.02438925 | 0.02610968 | 8.14E-04 | 0.07458272 | *Succinivibrionaceae* | BCA | Baseline (High-Potein) |
| 0.01365688 | 0.0192922 | 3.61E-05 | 0.05061714 | *Succinivibrionaceae* | BCA | Hydrolyzed |
| 0.03548605 | 0.03067571 | 3.14E-03 | 0.09407623 | *Succinivibrionaceae* | BCA | High-insoluble Fiber |
| 0.01493632 | 0.01989088 | 8.26E-05 | 0.05347657 | *Succinivibrionaceae* | BCA | Washout (High-Protein) |
| 0.02081248 | 0.02394666 | 4.04E-04 | 0.06735642 | *Enterobacteriaceae* | ACB | Baseline (High-Protein) |
| 0.02126522 | 0.02427655 | 4.32E-04 | 0.0688732 | *Enterobacteriaceae* | ACB | Hydrolyzed |
| 0.02679981 | 0.02722791 | 1.15E-03 | 0.07868504 | *Enterobacteriaceae* | ACB | High-insoluble Fiber |
| 0.04693586 | 0.03558848 | 6.66E-03 | 0.11363143 | *Enterobacteriaceae* | ACB | Washout (High-Protein) |
| 0.01622601 | 0.02099772 | 1.12E-04 | 0.05620067 | *Enterobacteriaceae* | BCA | Baseline (High-Protein) |
| 0.02384541 | 0.02555835 | 7.57E-04 | 0.07290881 | *Enterobacteriaceae* | BCA | Hydrolyzed |
| 0.01762941 | 0.02211543 | 1.76E-04 | 0.06035671 | *Enterobacteriaceae* | BCA | High-insoluble Fiber |
| 0.04891128 | 0.03626008 | 7.14E-03 | 0.11627848 | *Enterobacteriaceae* | BCA | Washout (High-Protein) |
| 0.03503384 | 0.03089517 | 2.98E-03 | 0.09369084 | OTHER | ACB | Baseline (High-Protein) |
| 0.03356335 | 0.02999081 | 2.57E-03 | 0.09038278 | OTHER | ACB | Hydrolyzed |
| 0.03335295 | 0.03025254 | 2.39E-03 | 0.09089613 | OTHER | ACB | High-insoluble Fiber |
| 0.03223247 | 0.02978768 | 2.23E-03 | 0.08935234 | OTHER | ACB | Washout (High-Protein) |
| 0.03157291 | 0.02901907 | 2.10E-03 | 0.08775944 | OTHER | BCA | Baseline (High-Protein) |
| 0.02826344 | 0.02777867 | 1.42E-03 | 0.08193567 | OTHER | BCA | Hydrolyzed |
| 0.03015389 | 0.02866466 | 1.75E-03 | 0.08530781 | OTHER | BCA | High-insoluble Fiber |
| 0.03016394 | 0.02872867 | 1.79E-03 | 0.08442404 | OTHER | BCA | Washout (High-Protein) |

Table 4B: Hierarchical Dirichlet regression model prediction values of the 'fitted' interval plots.

| **Estimate** | **Est.Error** | **Q5.5** | **Q94.5** | **Family** | **Sequence** | **Diet** |
| --- | --- | --- | --- | --- | --- | --- |
| 0.00778185 | 0.00136243 | 0.00579561 | 0.01013643 | *Bifidobacteriaceae* | ACB | Baseline (High-Protein) |
| 0.00839782 | 0.00161108 | 0.00601282 | 0.0111083 | *Bifidobacteriaceae* | ACB | Hydrolyzed |
| 0.03191253 | 0.00487924 | 0.0243595 | 0.03997478 | *Bifidobacteriaceae* | ACB | High-insoluble Fiber |
| 0.00926498 | 0.00177825 | 0.00662897 | 0.01230467 | *Bifidobacteriaceae* | ACB | Washout (High-Protein) |
| 0.00719875 | 0.00143371 | 0.00507681 | 0.00964168 | *Bifidobacteriaceae* | BCA | Baseline (High-Protein) |
| 0.01533038 | 0.00293102 | 0.01091367 | 0.02025512 | *Bifidobacteriaceae* | BCA | Hydrolyzed |
| 0.00818579 | 0.00165095 | 0.00573033 | 0.01098073 | *Bifidobacteriaceae* | BCA | High-insoluble Fiber |
| 0.0086365 | 0.00177693 | 0.00599274 | 0.01164674 | *Bifidobacteriaceae* | BCA | Washout (High-Protein) |
| 0.01893764 | 0.00334652 | 0.01387108 | 0.02452932 | *Coriobacteriaceae* | ACB | Baseline (High-Protein) |
| 0.03395845 | 0.00517959 | 0.02595001 | 0.04252929 | *Coriobacteriaceae* | ACB | Hydrolyzed |
| 0.02719084 | 0.00443159 | 0.02043794 | 0.03453497 | *Coriobacteriaceae* | ACB | High-insoluble Fiber |
| 0.02648278 | 0.00443329 | 0.01968079 | 0.03388418 | *Coriobacteriaceae* | ACB | Washout (High-Protein) |
| 0.01531736 | 0.00299157 | 0.01083522 | 0.02035721 | *Coriobacteriaceae* | BCA | Baseline (High-Protein) |
| 0.02935332 | 0.00492639 | 0.02185196 | 0.03760328 | *Coriobacteriaceae* | BCA | Hydrolyzed |
| 0.01882477 | 0.0035494 | 0.01342361 | 0.02471885 | *Coriobacteriaceae* | BCA | High-insoluble Fiber |
| 0.03734633 | 0.00583551 | 0.02843734 | 0.04706387 | *Coriobacteriaceae* | BCA | Washout (High-Protein) |
| 0.16032224 | 0.0145441 | 0.13773716 | 0.18429606 | *Bacteroidaceae* | ACB | Baseline (High-Protein) |
| 0.07569832 | 0.00956834 | 0.06092695 | 0.09134992 | *Bacteroidaceae* | ACB | Hydrolyzed |
| 0.04322896 | 0.00659526 | 0.03305389 | 0.05405071 | *Bacteroidaceae* | ACB | High-insoluble Fiber |
| 0.02604427 | 0.00458611 | 0.01913327 | 0.03375651 | *Bacteroidaceae* | ACB | Washout (High-Protein) |
| 0.17307192 | 0.01570491 | 0.14864708 | 0.19864455 | *Bacteroidaceae* | BCA | Baseline (High-Protein) |
| 0.02609208 | 0.00477413 | 0.01882672 | 0.03402549 | *Bacteroidaceae* | BCA | Hydrolyzed |
| 0.08889115 | 0.01081077 | 0.07224824 | 0.10675143 | *Bacteroidaceae* | BCA | High-insoluble Fiber |
| 0.03988507 | 0.0065926 | 0.02973274 | 0.0507981 | *Bacteroidaceae* | BCA | Washout (High-Protein) |
| 0.00624271 | 0.00126344 | 0.0043371 | 0.00837747 | *Prevotellaceae* | ACB | Baseline (High-Protein) |
| 0.00724519 | 0.0014812 | 0.00502177 | 0.00977225 | *Prevotellaceae* | ACB | Hydrolyzed |
| 0.03287517 | 0.00524792 | 0.02477386 | 0.04157352 | *Prevotellaceae* | ACB | High-insoluble Fiber |
| 0.00569405 | 0.00119429 | 0.00392475 | 0.00773314 | *Prevotellaceae* | ACB | Washout (High-Protein) |
| 0.00903716 | 0.00187628 | 0.00625456 | 0.01222946 | *Prevotellaceae* | BCA | Baseline (High-Protein) |
| 0.0061417 | 0.00131989 | 0.00418939 | 0.00837926 | *Prevotellaceae* | BCA | Hydrolyzed |
| 0.01408793 | 0.00281923 | 0.00987292 | 0.01879934 | *Prevotellaceae* | BCA | High-insoluble Fiber |
| 0.00553109 | 0.00119957 | 0.00375248 | 0.00758157 | *Prevotellaceae* | BCA | Washout (High-Protein) |
| 0.06585101 | 0.00802927 | 0.05328329 | 0.07909774 | *Paraprevotellaceae* | ACB | Baseline (High-Protein) |
| 0.07783752 | 0.00882658 | 0.06396231 | 0.09217498 | *Paraprevotellaceae* | ACB | Hydrolyzed |
| 0.04068522 | 0.00589301 | 0.03166784 | 0.05038957 | *Paraprevotellaceae* | ACB | High-insoluble Fiber |
| 0.02145604 | 0.00374417 | 0.01573778 | 0.0276516 | *Paraprevotellaceae* | ACB | Washout (High-Protein) |
| 0.0972821 | 0.01035648 | 0.08096877 | 0.11407946 | *Paraprevotellaceae* | BCA | Baseline (High-Protein) |
| 0.0413634 | 0.0063509 | 0.03163545 | 0.05181915 | *Paraprevotellaceae* | BCA | Hydrolyzed |
| 0.055725 | 0.00752684 | 0.04396842 | 0.0681835 | *Paraprevotellaceae* | BCA | High-insoluble Fiber |
| 0.03255839 | 0.00537974 | 0.02424749 | 0.04153636 | *Paraprevotellaceae* | BCA | Washout (High-Protein) |
| 0.00891274 | 0.00179309 | 0.00625546 | 0.01193739 | *Lactobacillaceae* | ACB | Baseline (High-Protein) |
| 0.01065171 | 0.00209155 | 0.00753935 | 0.01416114 | *Lactobacillaceae* | ACB | Hydrolyzed |
| 0.04051559 | 0.00631189 | 0.03105326 | 0.05105341 | *Lactobacillaceae* | ACB | High-insoluble Fiber |
| 0.01017876 | 0.00205621 | 0.00710774 | 0.01363708 | *Lactobacillaceae* | ACB | Washout (High-Protein) |
| 0.00978832 | 0.00205525 | 0.00671346 | 0.01325974 | *Lactobacillaceae* | BCA | Baseline (High-Protein) |
| 0.01542618 | 0.00307227 | 0.01081202 | 0.02060284 | *Lactobacillaceae* | BCA | Hydrolyzed |
| 0.01530206 | 0.00305361 | 0.01073329 | 0.02047546 | *Lactobacillaceae* | BCA | High-insoluble Fiber |
| 0.00931715 | 0.0019451 | 0.00639394 | 0.01257773 | *Lactobacillaceae* | BCA | Washout (High-Protein) |
| 0.00943472 | 0.00188825 | 0.0066073 | 0.01260667 | *Streptococcaceae* | ACB | Baseline (High-Protein) |
| 0.01816002 | 0.00335245 | 0.01312756 | 0.02383415 | *Streptococcaceae* | ACB | Hydrolyzed |
| 0.01855248 | 0.00337191 | 0.01345021 | 0.02420913 | *Streptococcaceae* | ACB | High-insoluble Fiber |
| 0.01162922 | 0.00230231 | 0.00820421 | 0.01553799 | *Streptococcaceae* | ACB | Washout (High-Protein) |
| 0.01136499 | 0.00234546 | 0.00786293 | 0.01532707 | *Streptococcaceae* | BCA | Baseline (High-Protein) |
| 0.01940146 | 0.00364355 | 0.01389546 | 0.02556056 | *Streptococcaceae* | BCA | Hydrolyzed |
| 0.01283309 | 0.00259397 | 0.0089603 | 0.01720165 | *Streptococcaceae* | BCA | High-insoluble Fiber |
| 0.01070646 | 0.00219224 | 0.00744724 | 0.01441946 | *Streptococcaceae* | BCA | Washout (High-Protein) |
| 0.01203518 | 0.00230831 | 0.00854145 | 0.01591136 | *Turicibacteraceae* | ACB | Baseline (High-Protein) |
| 0.02228459 | 0.0038713 | 0.01639033 | 0.0288047 | *Turicibacteraceae* | ACB | Hydrolyzed |
| 0.1040542 | 0.01011713 | 0.08810057 | 0.12056321 | *Turicibacteraceae* | ACB | High-insoluble Fiber |
| 0.01154127 | 0.00225882 | 0.00813871 | 0.01533926 | *Turicibacteraceae* | ACB | Washout (High-Protein) |
| 0.0132774 | 0.00265366 | 0.0092485 | 0.01773768 | *Turicibacteraceae* | BCA | Baseline (High-Protein) |
| 0.03008268 | 0.00501658 | 0.02241066 | 0.03846128 | *Turicibacteraceae* | BCA | Hydrolyzed |
| 0.04643826 | 0.00670521 | 0.03610008 | 0.05752614 | *Turicibacteraceae* | BCA | High-insoluble Fiber |
| 0.01042782 | 0.00215003 | 0.00719066 | 0.01401236 | *Turicibacteraceae* | BCA | Washout (High-Protein) |
| 0.08303133 | 0.00939012 | 0.06830054 | 0.09832591 | *Clostridiaceae* | ACB | Baseline (High-Protein) |
| 0.09381779 | 0.00999846 | 0.07793298 | 0.10996096 | *Clostridiaceae* | ACB | Hydrolyzed |
| 0.09955943 | 0.01015121 | 0.083674 | 0.11599001 | *Clostridiaceae* | ACB | High-insoluble Fiber |
| 0.21158959 | 0.01486456 | 0.18835114 | 0.23558968 | *Clostridiaceae* | ACB | Washout (High-Protein) |
| 0.05421471 | 0.00755793 | 0.04256038 | 0.0666394 | *Clostridiaceae* | BCA | Baseline (High-Protein) |
| 0.14957423 | 0.01304673 | 0.12914738 | 0.17062131 | *Clostridiaceae* | BCA | Hydrolyzed |
| 0.0750673 | 0.00915672 | 0.06068976 | 0.0899183 | *Clostridiaceae* | BCA | High-insoluble Fiber |
| 0.18423717 | 0.01439428 | 0.16129775 | 0.20745623 | *Clostridiaceae* | BCA | Washout (High-Protein) |
| 0.10919877 | 0.01042449 | 0.09282775 | 0.12613203 | *Lachnospiraceae* | ACB | Baseline (High-Protein) |
| 0.12909002 | 0.01138378 | 0.11092024 | 0.14755842 | *Lachnospiraceae* | ACB | Hydrolyzed |
| 0.14637524 | 0.01185055 | 0.12755239 | 0.16552985 | *Lachnospiraceae* | ACB | High-insoluble Fiber |
| 0.0907084 | 0.00950656 | 0.07571484 | 0.10597432 | *Lachnospiraceae* | ACB | Washout (High-Protein) |
| 0.10822352 | 0.01099824 | 0.09096995 | 0.12606086 | *Lachnospiraceae* | BCA | Baseline (High-Protein) |
| 0.18208846 | 0.013946 | 0.16021099 | 0.20458397 | *Lachnospiraceae* | BCA | Hydrolyzed |
| 0.12741477 | 0.01182446 | 0.10900481 | 0.14661105 | *Lachnospiraceae* | BCA | High-insoluble Fiber |
| 0.11465121 | 0.0112768 | 0.09698047 | 0.13313474 | *Lachnospiraceae* | BCA | Washout (High-Protein) |
| 0.01869919 | 0.00335765 | 0.01364762 | 0.02428316 | *Peptococcaceae* | ACB | Baseline (High-Protein) |
| 0.03588099 | 0.00544512 | 0.02761529 | 0.04486098 | *Peptococcaceae* | ACB | Hydrolyzed |
| 0.01999679 | 0.00346788 | 0.01469374 | 0.02575699 | *Peptococcaceae* | ACB | High-insoluble Fiber |
| 0.01907976 | 0.00340991 | 0.01388184 | 0.0247343 | *Peptococcaceae* | ACB | Washout (High-Protein) |
| 0.02089316 | 0.00382314 | 0.01503806 | 0.02732529 | *Peptococcaceae* | BCA | Baseline (High-Protein) |
| 0.03597392 | 0.00564824 | 0.02727469 | 0.04529657 | *Peptococcaceae* | BCA | Hydrolyzed |
| 0.01548063 | 0.00303863 | 0.01094808 | 0.0205739 | *Peptococcaceae* | BCA | High-insoluble Fiber |
| 0.02605228 | 0.00461763 | 0.01896543 | 0.03372847 | *Peptococcaceae* | BCA | Washout (High-Protein) |
| 0.03984338 | 0.00601975 | 0.03056976 | 0.04976644 | *Peptostreptococcaceae* | ACB | Baseline (High-Protein) |
| 0.02492048 | 0.00428505 | 0.01840338 | 0.03207764 | *Peptostreptococcaceae* | ACB | Hydrolyzed |
| 0.03495598 | 0.00548209 | 0.02652257 | 0.04404289 | *Peptostreptococcaceae* | ACB | High-insoluble Fiber |
| 0.09628871 | 0.01034932 | 0.08038145 | 0.11356529 | *Peptostreptococcaceae* | ACB | Washout (High-Protein) |
| 0.02422508 | 0.00437211 | 0.0175726 | 0.03145711 | *Peptostreptococcaceae* | BCA | Baseline (High-Protein) |
| 0.02868287 | 0.0049351 | 0.02121116 | 0.03700649 | *Peptostreptococcaceae* | BCA | Hydrolyzed |
| 0.02644077 | 0.00464275 | 0.01930831 | 0.03423233 | *Peptostreptococcaceae* | BCA | High-insoluble Fiber |
| 0.0771501 | 0.00951544 | 0.06243338 | 0.09287696 | *Peptostreptococcaceae* | BCA | Washout (High-Protein) |
| 0.03003782 | 0.00480874 | 0.02262687 | 0.03797625 | *Ruminococcaceae* | ACB | Baseline (High-Protein) |
| 0.04310745 | 0.00613027 | 0.03356214 | 0.05306437 | *Ruminococcaceae* | ACB | Hydrolyzed |
| 0.05091003 | 0.006808 | 0.04023566 | 0.06199178 | *Ruminococcaceae* | ACB | High-insoluble Fiber |
| 0.01649089 | 0.0030541 | 0.01192448 | 0.02160778 | *Ruminococcaceae* | ACB | Washout (High-Protein) |
| 0.04079794 | 0.00618025 | 0.03120676 | 0.05107637 | *Ruminococcaceae* | BCA | Baseline (High-Protein) |
| 0.04162964 | 0.00622046 | 0.03208943 | 0.05183592 | *Ruminococcaceae* | BCA | Hydrolyzed |
| 0.06128408 | 0.00792552 | 0.04895643 | 0.07421932 | *Ruminococcaceae* | BCA | High-insoluble Fiber |
| 0.02061545 | 0.00380117 | 0.01487583 | 0.02697813 | *Ruminococcaceae* | BCA | Washout (High-Protein) |
| 0.049053 | 0.00672557 | 0.03863293 | 0.06004194 | *Veillonellaceae* | ACB | Baseline (High-Protein) |
| 0.1141831 | 0.01080508 | 0.09723976 | 0.13177562 | *Veillonellaceae* | ACB | Hydrolyzed |
| 0.08894321 | 0.00945617 | 0.07422248 | 0.10446198 | *Veillonellaceae* | ACB | High-insoluble Fiber |
| 0.02198132 | 0.00381291 | 0.01615836 | 0.02837944 | *Veillonellaceae* | ACB | Washout (High-Protein) |
| 0.07692329 | 0.00919144 | 0.06235463 | 0.09182875 | *Veillonellaceae* | BCA | Baseline (High-Protein) |
| 0.09472375 | 0.01025334 | 0.07864356 | 0.11142628 | *Veillonellaceae* | BCA | Hydrolyzed |
| 0.14560802 | 0.01267059 | 0.12566355 | 0.16596485 | *Veillonellaceae* | BCA | High-insoluble Fiber |
| 0.02915951 | 0.00497211 | 0.02151964 | 0.03739363 | *Veillonellaceae* | BCA | Washout (High-Protein) |
| 0.01268291 | 0.00243589 | 0.00898499 | 0.01679352 | *Tissierellaceae* | ACB | Baseline (High-Protein) |
| 0.00913397 | 0.00184498 | 0.00640178 | 0.01223895 | *Tissierellaceae* | ACB | Hydrolyzed |
| 0.00950059 | 0.00187917 | 0.00669533 | 0.01264313 | *Tissierellaceae* | ACB | High-insoluble Fiber |
| 0.02969496 | 0.0050116 | 0.02215638 | 0.03805878 | *Tissierellaceae* | ACB | Washout (High-Protein) |
| 0.01067702 | 0.00222618 | 0.00739903 | 0.01448176 | *Tissierellaceae* | BCA | Baseline (High-Protein) |
| 0.00827608 | 0.00174295 | 0.00567566 | 0.01124099 | *Tissierellaceae* | BCA | Hydrolyzed |
| 0.00864353 | 0.00182454 | 0.00591744 | 0.01172736 | *Tissierellaceae* | BCA | High-insoluble Fiber |
| 0.03022202 | 0.00521875 | 0.02226424 | 0.03885929 | *Tissierellaceae* | BCA | Washout (High-Protein) |
| 0.01680166 | 0.00306285 | 0.01217984 | 0.02199303 | *Erysipelotrichaceae* | ACB | Baseline (High-Protein) |
| 0.01776487 | 0.00323861 | 0.01284605 | 0.02316079 | *Erysipelotrichaceae* | ACB | Hydrolyzed |
| 0.03731402 | 0.0055838 | 0.02868724 | 0.04654447 | *Erysipelotrichaceae* | ACB | High-insoluble Fiber |
| 0.02004303 | 0.00350379 | 0.01467718 | 0.02589186 | *Erysipelotrichaceae* | ACB | Washout (High-Protein) |
| 0.01770028 | 0.0033773 | 0.01262301 | 0.02339549 | *Erysipelotrichaceae* | BCA | Baseline (High-Protein) |
| 0.02138023 | 0.00391549 | 0.01538614 | 0.02785967 | *Erysipelotrichaceae* | BCA | Hydrolyzed |
| 0.02188285 | 0.00392949 | 0.0158939 | 0.02840256 | *Erysipelotrichaceae* | BCA | High-insoluble Fiber |
| 0.01995956 | 0.00368932 | 0.01434019 | 0.026121 | *Erysipelotrichaceae* | BCA | Washout (High-Protein) |
| 0.23638029 | 0.01614902 | 0.21075206 | 0.26254983 | *Fusobacteriaceae* | ACB | Baseline (High-Protein) |
| 0.17126571 | 0.01399593 | 0.14936709 | 0.1939346 | *Fusobacteriaceae* | ACB | Hydrolyzed |
| 0.05740023 | 0.00747955 | 0.04578013 | 0.06972085 | *Fusobacteriaceae* | ACB | High-insoluble Fiber |
| 0.26056813 | 0.01655211 | 0.23428856 | 0.28685 | *Fusobacteriaceae* | ACB | Washout (High-Protein) |
| 0.20738339 | 0.01591317 | 0.18210328 | 0.2333708 | *Fusobacteriaceae* | BCA | Baseline (High-Protein) |
| 0.16491894 | 0.01426058 | 0.14247149 | 0.18791675 | *Fusobacteriaceae* | BCA | Hydrolyzed |
| 0.15201473 | 0.01366099 | 0.13053078 | 0.17407815 | *Fusobacteriaceae* | BCA | High-insoluble Fiber |
| 0.23025103 | 0.01647329 | 0.20461672 | 0.25720587 | *Fusobacteriaceae* | BCA | Washout (High-Protein) |
| 0.03458279 | 0.00530936 | 0.02643115 | 0.04338751 | *Alcaligenaceae* | ACB | Baseline (High-Protein) |
| 0.03058058 | 0.00487654 | 0.0230478 | 0.03873437 | *Alcaligenaceae* | ACB | Hydrolyzed |
| 0.03781534 | 0.00560253 | 0.02912299 | 0.04711923 | *Alcaligenaceae* | ACB | High-insoluble Fiber |
| 0.01855538 | 0.00338757 | 0.01342265 | 0.02424777 | *Alcaligenaceae* | ACB | Washout (High-Protein) |
| 0.0301694 | 0.00505872 | 0.02245084 | 0.0386076 | *Alcaligenaceae* | BCA | Baseline (High-Protein) |
| 0.02367891 | 0.00416274 | 0.0173756 | 0.03052214 | *Alcaligenaceae* | BCA | Hydrolyzed |
| 0.02292685 | 0.00411082 | 0.01664344 | 0.02985601 | *Alcaligenaceae* | BCA | High-insoluble Fiber |
| 0.01888121 | 0.00351299 | 0.01355284 | 0.02477223 | *Alcaligenaceae* | BCA | Washout (High-Protein) |
| 0.02427167 | 0.00410744 | 0.01799387 | 0.0311815 | *Succinivibrionaceae* | ACB | Baseline (High-Protein) |
| 0.0212989 | 0.00371721 | 0.01563988 | 0.02755052 | *Succinivibrionaceae* | ACB | Hydrolyzed |
| 0.01887701 | 0.003395 | 0.01372079 | 0.02458581 | *Succinivibrionaceae* | ACB | High-insoluble Fiber |
| 0.01328081 | 0.0025474 | 0.00943805 | 0.01757453 | *Succinivibrionaceae* | ACB | Washout (High-Protein) |
| 0.02441136 | 0.00438729 | 0.01775765 | 0.03176784 | *Succinivibrionaceae* | BCA | Baseline (High-Protein) |
| 0.01358011 | 0.00270321 | 0.00953859 | 0.01811847 | *Succinivibrionaceae* | BCA | Hydrolyzed |
| 0.0355141 | 0.00563703 | 0.02678789 | 0.04485765 | *Succinivibrionaceae* | BCA | High-insoluble Fiber |
| 0.01517263 | 0.00294597 | 0.01079655 | 0.02022697 | *Succinivibrionaceae* | BCA | Washout (High-Protein) |
| 0.02088204 | 0.00370767 | 0.0152396 | 0.02710555 | *Enterobacteriaceae* | ACB | Baseline (High-Protein) |
| 0.02107623 | 0.00371009 | 0.01540881 | 0.02729758 | *Enterobacteriaceae* | ACB | Hydrolyzed |
| 0.02647563 | 0.00446876 | 0.0196687 | 0.033931 | *Enterobacteriaceae* | ACB | High-insoluble Fiber |
| 0.04724566 | 0.00656351 | 0.03711549 | 0.0579875 | *Enterobacteriaceae* | ACB | Washout (High-Protein) |
| 0.01625867 | 0.00318954 | 0.01146826 | 0.0216509 | *Enterobacteriaceae* | BCA | Baseline (High-Protein) |
| 0.02396285 | 0.00427613 | 0.01751325 | 0.03115697 | *Enterobacteriaceae* | BCA | Hydrolyzed |
| 0.01735826 | 0.0033358 | 0.01230079 | 0.02302666 | *Enterobacteriaceae* | BCA | High-insoluble Fiber |
| 0.0489238 | 0.00703506 | 0.03799373 | 0.0604644 | *Enterobacteriaceae* | BCA | Washout (High-Protein) |
| 0.03501707 | 0.00533735 | 0.02665205 | 0.04374686 | OTHER | ACB | Baseline (High-Protein) |
| 0.03364629 | 0.00517386 | 0.02565683 | 0.0420887 | OTHER | ACB | Hydrolyzed |
| 0.0328615 | 0.00506746 | 0.02508702 | 0.0413259 | OTHER | ACB | High-insoluble Fiber |
| 0.03218198 | 0.00506109 | 0.02430956 | 0.04055568 | OTHER | ACB | Washout (High-Protein) |
| 0.03178421 | 0.0051421 | 0.02387905 | 0.04033051 | OTHER | BCA | Baseline (High-Protein) |
| 0.02833882 | 0.00474678 | 0.02104009 | 0.03628947 | OTHER | BCA | Hydrolyzed |
| 0.03007606 | 0.00504104 | 0.02237813 | 0.03841696 | OTHER | BCA | High-insoluble Fiber |
| 0.03031523 | 0.00510778 | 0.02243248 | 0.03881845 | OTHER | BCA | Washout (High-Protein) |
